# Lytic coelomocyte death is tuned by cleavage but not phosphorylation of MLKL in echinoderms

**DOI:** 10.1371/journal.ppat.1012991

**Published:** 2025-03-14

**Authors:** Kaiyu Chen, Sikou Shen, Zhimeng Lv, Ming Guo, Yina Shao, Chenghua Li

**Affiliations:** 1 State Key Laboratory of Agricultural Products Safety, Ningbo University, Ningbo, People's Republic of China; 2 Laboratory for Marine Fisheries Science and Food Production Processes, Qingdao National Laboratory for Marine Science and Technology, Qingdao, People's Republic of China; Uppsala University, SWEDEN

## Abstract

Lytic cell death including necroptosis and pyroptosis is induced by mixed lineage kinase domain-like protein (MLKL) phosphorylation and inflammatory caspase specific cleavage Gasdermins in higher mammals, respectively. In this study, we identified a novel MLKL homolog containing a tetrapeptide recognition motif (14-LVAD-17) of inflammatory caspase from *Apostichopus japonicus*,which was absent of Gasdermins member by genome screening. Functional analysis revealed that AjMLKL was involved in the regulation of *Vibrio splendidus* AJ01 infection induced lytic coelomocyte death in a cleavage-dependent manner, but not through RIPK3-dependent phosphorylation as mammals. Mechanistically, the activated form of cysteine-aspartic specific proteases-1 (AjCASP-1) bound to the tetrapeptide site of AjMLKL and cleaved it at Asp^17^. Cleaved AjMLKL^18-491^ displayed higher binding affinities towards phosphatidylinositol phosphate and cardiolipin compared to those of un-cleaved form. In addition, cleaved AjMLKL^18-491^ exerted stronger ability in disrupting the membrane integrity of liposome. More importantly, AjMLKL^18-491^ caused a large non-selective ionic coelomocyte pore and could directly kill the invasive AJ01. Moreover, activation of inflammatory AjCASP-1 was further found to be dependent on forming an inflammasome-like complex via CASc domain of AjCASP-1 and the N-terminal Ig domains of internalized AjNLRC4. All our results proved first evidence that lytic cell death was activated through MLKL cleavage, not MLKL phosphorylation in echinoderm, which offered insights into the functional, evolutionary mechanisms of lytic cell death in invertebrates.

## Author summary

Lytic cell death, as a new type of programmed cell death, is the gatekeeper of host defense against certain pathogen invasions. As a known terminal effector in the process of lytic cell death in mammals, MLKL has a very different activation mechanism in different species. However, how it regulates lytic cell death in invertebrates is still under debate. In this study, we report a novel mechanism of MLKL cleavage activation in the sea cucumber to regulate lytic cell death, which relies on the CARD domain-independent assembly mode of inflammasome and the formation of large pore size non-selective ion channels in the cell membrane by the cleaved MLKL. This means that in sea cucumber which lack the key pyroptosis protein gasdermins, MLKL activated in this way may play an important role in antibacterial infection as an alternative of pyroptosis. Meanwhile, the results of our study unveil the presence of functional MLKL in Echinoderms and provide insights into the biological role and evolution of invertebrate MLKL.

## Introduction

Lytic cell death and apoptosis are both programmed cell death that play fundamental role in innate immunity from plants and animals [1–3], which is characterized by different morphological and biochemical changes associated with losing of cell vitality. Apoptotic cell death is considered as a non-inflammatory process that eliminates damaged or infected cells predominantly in an immunologically silent manner [1]. On the contrary, lytic cell death causes increased permeability of plasma membrane and leads to the release of inflammatory cellular contents, including damage-associated molecular patterns (DAMPs) and inflammatory cytokines such as interleukin-1β (IL-1β) to self-amplify or even trigger the inflammatory process [4]. Lytic cell death plays a pivotal host defense response against invading pathogens and its molecular mechanism has been intensively investigated [5–9]. Necrosomes formed after necroptotic stimulation play a key role in initiating necroptosis [10–12]. Proteins involves in the regulation of lytic cell death from plants, animals, and fungi share a conserved four-helix bundle structure called the 4HB (four-helix bundle) domain [13]. mixed lineage kinase domain-like protein (MLKL) is composed of an internal coiled coil (CC) region, a C-terminal kinase like domain, as well as a N-terminal 4HB domain, which acts as terminal effector protein to execute necroptosis. During necroptosis, Thr357/Ser358 sites on the kinase like domain of MLKL is phosphorylated by receptor-interacting serine/threonine-protein kinase 3 (RIPK3), before translocating to the plasma membrane where it disrupts membrane integrity [14–16].

Recent studies have revealed distinct mechanistic differences in MLKL activation during necroptosis among different species [17–21], in which the recognition and phosphorylation of MLKL by RIPK3 is highly-speciated [20]. For instance, even though rat and mouse MLKL protein share >85% in their sequence identity, rat MLKL is unable to reconstitute the necroptosis pathway in mouse cells lacking MLKL [20]. In mouse cells, the executioner function of the 4HB domain is repressed by MLKL pseudo-kinase domain until RIPK3-mediated phosphorylation relieves this interaction and triggers 4HB-directed cell death [14]. By contrast, in human cells, phosphorylation alone appears insufficient for MLKL activation [17,22]. Instead, activation of human MLKL relies on a stable interaction with RIPK3 [17,20,21]. Despite of the well-documented phosphorylation of MLKL, cleavage of MLKL at Asp140 in the 137-DQQD-140 (D:Asp, Q:Gln) by cysteine-aspartic specific proteases (CASP) -3/8/10 is also found to induce necroptosis in a RIPK3-independent manner from human myeloma (MM) cells [23]. However, it remains less understood and disputed on the molecular mechanisms of cleaved MLKL-mediated necroptosis.

Despite the research efforts on lytic cell death from human and mouse, evidence from multiple model systems have extended the existence of lytic cell death in some model systems, including *Caenorhabditis elegans* [24], *Dictyostelium discoideum* [25], *Saccharomyces cerevisiae* [26], and *Drosophila melanogaster* [27]. However, most of these studies are still focus on descriptive and morphological characterization, while the molecular participators and mechanisms remain less explored [28,29]. It has been reported that several components of the lytic cell death pathway such as RIPK3 and MLKL are surprisingly poorly conserved within the animal kingdom, which suggests that RIPK3–MLKL-mediated necroptosis, as we know it in humans and mice, cannot be a universal cell death pathway [30]. In the present study, we detected lytic coelomocyte death in sea cucumber *Apostichopus japonicus* during *Vibrio splendidus* AJ01 infection, which was mediated by a unique activation mechanism of AjMLKL through AjCaspase-1 (AjCASP-1)-dependent cleavage. We also demonstrated that intracellular AjNLRC4 could form inflammasome-like complex via interacting with AjCASP-1 and further activated AjCASP-1 to p20/p10 forms. Interestingly, in mammals, another key executive protein of lytic cell death, Gasdermin D (GSDMD), is activated and cleaved by the inflammasome pathway. However, sea cucumbers lack the GSDM protein family, and AjMLKL replaces GSDM to regulate sea cucumber lytic cell death in a manner similar to mammalian inflammasome activation and cleavage. This may represent an unexpected selection in the evolutionary process, suggesting that the processing of MLKL by “early inflammasomes” is an evolutionary precursor to true GSDM-mediated cell pyroptosis in higher organisms. It also means that pyroptosis and necroptosis may have merged at a certain stage of evolution. All these results enhanced our present understanding of the evolution, function, and activation mechanism of lytic cell death.

## Results

### AJ01 infection at 10^8^ CFU/mL induces lytic coelomocyte death in sea cucumbers

**Fig 1:**
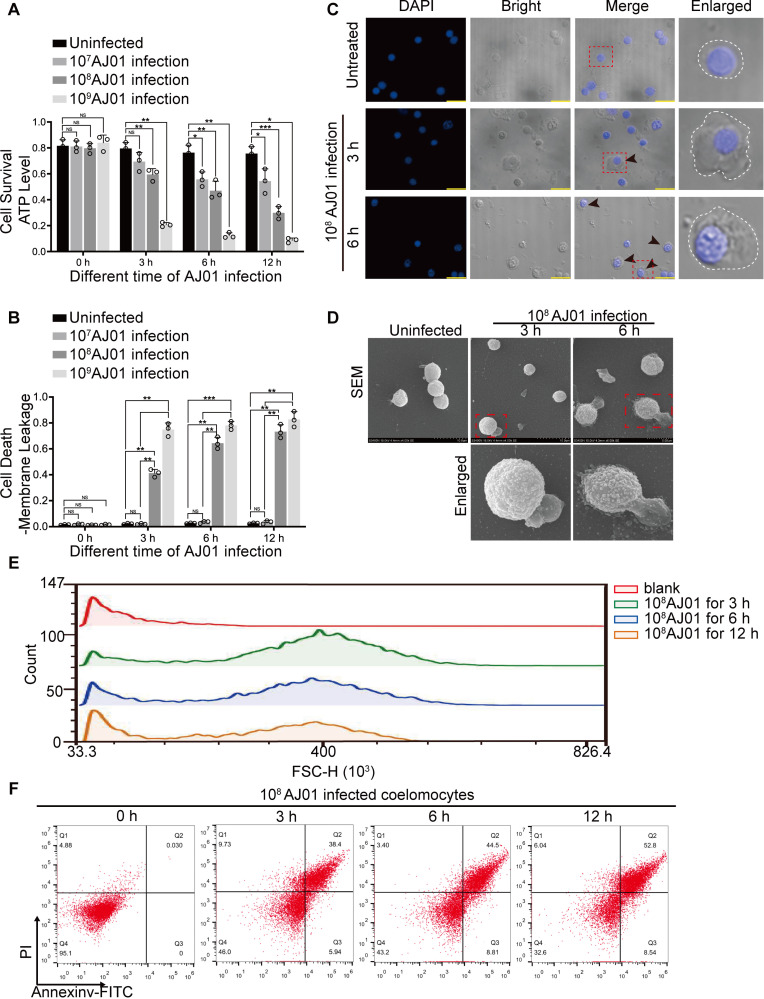
High concentration of *V. splendidus* AJ01 infection induces coelomocytes lytic coelomocyte death. (A) Sea cucumbers were immersed with different concentrations of AJ01 (10^7^, 10^8^, and 10^9^ CFU/mL) for 0, 3, 6, and 12 h. Sea cucumbers without any treatment served as the controls. Coelomocytes and coelomic fluid supernatants were collected from infection and control groups. 10^4^ coelomocytes were collected from each group, and ATP levels were measured using the CellTiter-Glo kit to determine coelomocyte viability. (B) The collected coelomic fluid supernatants were analyzed for coelomocyte plasma membrane disruption using the CytoTox-Glo cytotoxicity assay kit. (C and D) The morphology of coelomocytes was analyzed using confocal microscopy. Scale bar, 5 μm (C) and FEG-SEM. Dashed lines represent cell membrane boundaries (D), Arrows indicate cells exhibiting necrotic-like features, The dotted lines represent the boundaries of the cell membrane. (E) 10^8^ CFU/mL AJ01 infected coelomocytes for 3, 6 and 12 hours, the forward scatter (FSC) of intact cells was assessed by flow cytometry. (F) The propidium iodide and Annexin V-fluorescein isothiocyanate (FITC)-stained coelomocytes were further analyzed by flow cytometry. All data are plotted as mean ± SEM, asterisks indicate significant differences: *p < 0.05, **p < 0.01, ***p < 0.001.

Necroptotic cell death and pyroptosis were characterized as lytic cell death with necrotic morphologies such as rupture of plasma membrane and cell swelling [31]. To investigate whether lytic coelomocyte death was existed in sea cucumber, we treated sea cucumber with different concentrations of AJ01 and cell membrane leakage, ATP drop were analyzed at 0, 3, 6, and 12 h. The results indicated there were no significant change in ATP drop (81.62 ± 4.68%–75.58 ± 5.32%), and no cell membrane leakage at different time points in the control group (Fig 1A and 1B). AJ01 treatment showed that coelomocyte ATP level significant dropped from 81.01 ± 2.45% to 54.4 ± 9.46%, while no significant leakage of the cytoplasmic membrane was detected at all examined time points in 10^7^ CFU/mL AJ01 group (Fig 1A and 1B). When the dose of AJ01 increased to 10^8^ CFU/mL, coelomocyte ATP dropped from 79.68 ± 3.84% to 29.85 ± 4.83% after 12 hours (Fig 1A), while the membrane leakage was significantly increased from 1.21 ± 0.19% to 73.29 ± 5.31% (Fig 1B). The changes in coelomocyte ATP and membrane leakage were accelerated under 10^9^ CFU/mL AJ01 treatment, which ATP was dropped from 83.95 ± 5.89% to 8.69 ± 1.77% and membrane leakage was increased from 1.46 ± 0.46% to 81.80 ± 4.65%, respectively (Fig 1A and 1B). Regarding the low coelomocyte viability at 10^9^ CFU/mL group, we selected 10^8^ CFU/mL as a proper dose for subsequent lytic cell death characterization. By using a laser scanning confocal microscopy, we could clearly see coelomocytes with obvious lytic cell death morphologies including the bubble-like membrane structures and cell swelling (Fig 1C), which were also confirmed by a FEG-SEM (Fig 1D). We then performed flow cytometry analysis to measure cell swelling by using forward scatter measurements to quantify cell size according to previous reports [32–35]. A proportion of coelomocytes with increased forward scatter was detected from 3 h to 12h post AJ01 treatment compared to that of untreated group (Fig 1E). AJ01 treatment increased the percentage of Annexin V+/PI+ coelomocyte population compared with that of untreated group (Fig 1F). In addition, the percentage of the Annexin V+/PI+ population was significantly larger than those of the Annexin V+/PI− and Annexin V−/PI+ populations (Fig 1F) from AJ01 group. All these results indicated 10^8^ CFU/mL AJ01 infection could significantly induce lytic coelomocyte death of sea cucumber.

### AjMLKL is involved in AJ01 induced lytic coelomocyte death independent of phosphorylation

**Fig 2:**
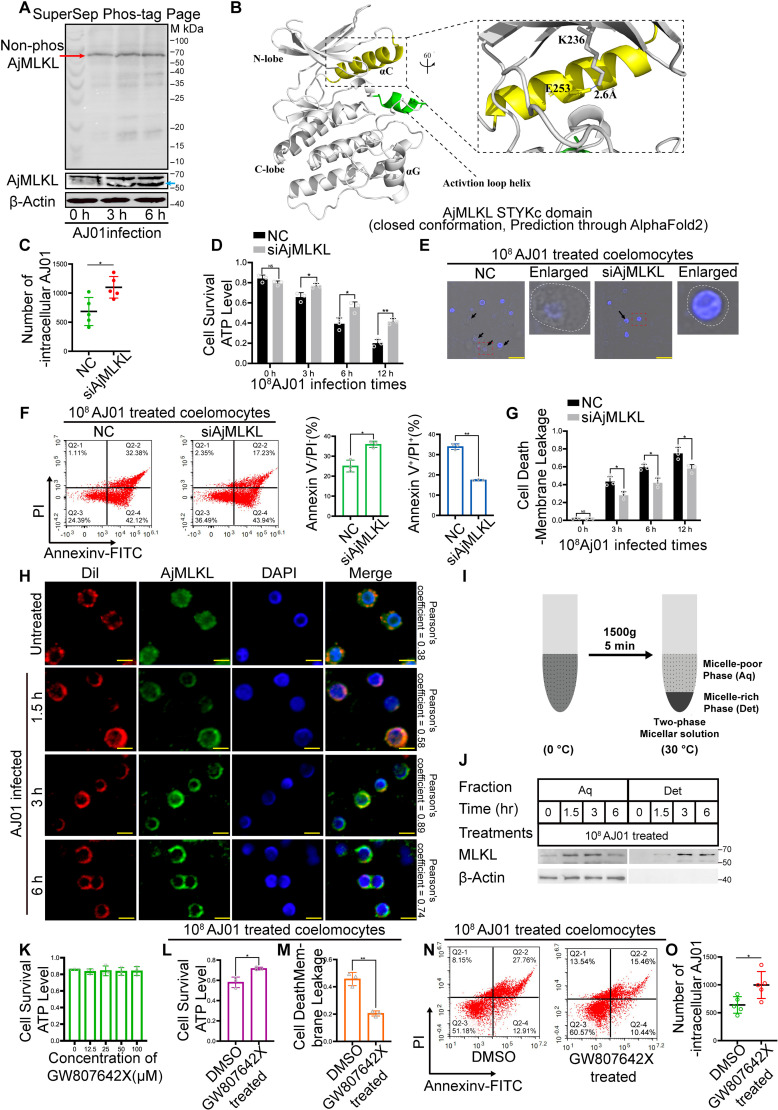
AjMLKL is involved in 10^8^ CFU/mL AJ01 induced lytic coelomocyte death independent on its phosphorylation. (A) Expression patterns of AjMLKL protein and phosphorylation levels in coelomocytes after 10^8^ CFU/ml AJ01 challenge as detected using western blotting (Lower) with β-Actin as the loading control. The red arrow represents unphosphorylated AjMLKL, and the blue arrow represents the lower band of AjMLKL. (B) The predicted structure of AjMLKL- STYKc domain adopts a closed conformation with an intact VVIK-αC salt bridge (zoomed panel). (C-D) To confirm AjMLKL involved in AJ01 infection-induced lytic coelomocytes death in sea cucumber coelomocytes, the number of intracellular AJ01 survival (C), coelomocyte viability (D), the number of coelomocytes with lytic morphology, Scale bar, 10 μm (E), the proportion of Annexin V+/PI+ lytic coelomocytes (F) and the coelomocytes plasma membrane disruption (G) were assayed in 10^8^ CFU/mL AJ01-challenged AjMLKL silenced coelomocytes for different times. (H) To address the AjMLKL translocates to the membrane fraction during lytic coelomocytes death, The migration of AjMLKL to the cell membrane was detected at 0, 1.5, 3 and 6 h after AJ01 stimulation through immunocytochemistry. (I) A schematic representation of the phase separation of integral membrane proteins in Trion X-114 solution. (J) AjMLKL and a smaller band of AjMLKL were concentrated in the micelle-rich fraction after phase separation. coelomocytes were treated with AJ01 for the indicated time. The cells were harvested and solubilized in Triton X-114 lysis buffer then separated into aqueous phase (Aq) and detergent phase (Det) as described in the Experimental Procedures and as shown in (I). The samples were analyzed by western blotting using antibodies as indicated. β-actin was shown as loading controls. (K-O) Commercial MLKL-specific inhibitor GW806724X was further used. The 100 μM GW806724X treatment did not reduce coelomocyte viability (K). Treatment of sea cucumber coelomocytes with 100 μM GW806724X for 3 h and then challenged by 10^8^ CFU/mL AJ01. Coelomocyte viability was significantly increased (L), and coelomocytes plasma membrane disruption (M) and the percentage of Annexin V+/PI+ coelomocytes significantly decreased (N). Intracellular AJ01 survival significantly increased in the GW806724X-treated group (O). All data are plotted as mean ± SEM, asterisks indicate significant differences: *p < 0.05, **p < 0.01, ***p < 0.001.

MLKL is the core effector of necroptosis through its phosphorylation by the RIPK3, which leads to the translocation of MLKL to cell membrane to induce necroptotic cell death in vertebrates [36–39]. By using Genscan and BLAST programs with full-length sequences of MLKLs from *Homo sapiens* and *Mus musculus* as queries, we were able to retrieve a MLKL gene homolog (AjMLKL, PIK49054.1) from sea cucumber genome and expressed sequence tags databases (S1A–S1C Fig). A specific AjMLKL antibody was prepared and its specificity was confirmed (S1D Fig), we next examined the expression levels of AjMLKL protein and phosphorylation in coelomocytes after AJ01 treatment. Western blot analysis revealed that AJ01 treatment induced a smaller band of AjMLKL protein with an approximate molecular weight of 53 KDa compared to the single band at untreated group (Figs 2Aand S1E). Since the level of phosphorylated MLKL is considered as the core detection index of necroptosis [40], we ran a phosphor-labeled gel to detect the phosphorylation status of AjMLKL [41]. No obvious band shift was observed from the total protein of coelomocyte after AJ01 infection (Fig 2A). This suggests that AjMLKL may not need to respond to AJ01 infection in a phosphorylation-dependent manner. Consistently, the interaction of AjMLKL with AjRIPK3 was not detected by Co-IP assay (S2A–S2D Fig). This implies that the RIPK3 phosphorylation of MLKL, a pathway conserved in mammals that regulates lytic cell death, is not conserved in sea cucumber. Previously, It has been reported that the MLKL pseudokinase domain undergoes a conformational change, adopting a closed, active kinase-like conformation upon RIPK3-mediated phosphorylation [42]. To further explore the reason why AjMLKL does not undergo phosphorylation, we predicted the STYKc domain of AjMLKL through AlphaFold2 [43]. Interestingly, the STYKc domain of AjMLKL directly adopts a closed conformation, exhibiting classical active kinase features including a salt bridge between the β3 strand Lys (VVIK236) and the αC helix Glu (E253) (Fig 2B). These implied that the kinase domain of AjMLKL is directly characterised by a closed active kinase-like conformation, rendering it unnecessary for additional phosphorylation modifications.

To further validate the potential role of AjMLKL in AJ01 induced lytic coelomocytes death, we performed RNAi-mediated AjMLKL knockdown by using a specific siRNA (S1 Table). Protein and mRNA expression levels of AjMLKL were significantly downregulated after AjMLKL siRNA transfection (S2E–S2F Fig). We next challenged the coelomocytes with 10^8^ CFU/mL AJ01 and the results revealed that AjMLKL knockdown significantly increased the number of intracellular AJ01 (Fig 2C) and coelomocyte viability (Fig 2D). Coelomocytes with lytic morphology were also reduced in AjMLKL siRNA transfection group compared with those of NC group (Fig 2E). Knockdown of AjMLKL also decreased the proportion of Annexin V+/PI+ coelomocytes (Fig 2F) and the cell membrane leakage (Fig 2G). All these results indicated unphosphorylated AjMLKL was involved in lytic coelomocyte death upon AJ01 infection.

To further determine the translocation of AjMLKL in response to AJ01 infection, we performed immunocytochemistry to detect the subcellular localization of AjMLKL using an anti-AjMLKL antibody. Under normal conditions, AjMLKL was mainly located on the cytoplasm (Fig 2H), while after 10^8^CFU/ml AJ01 infection AjMLKL signal was colocalized with DiI, which was a specific indicator of the cell membrane (Fig 2H). To further characterize this process, we took advantage of a unique property of a detergent, Triton X-114, that undergoes phase transition at different temperatures [38]. As illustrated in Fig 2I, when we used Triton X-114 to extract coelomocytes undergoing AJ01 infection at different time points and subjected the coelomocytes extracts to the temperature-dependent phase separation procedure, we observed AjMLKL moved from the aqueous phase to detergent phase after AJ01 infection for 1.5 h. Interestingly, two AjMLKL products also appeared in the detergent phase at this time (Fig 2J).

We further validated lytic coelomocytes death was dependent on the membrane transport process of AjMLKL by using a commercial MLKL-specific inhibitor GW806742X, which targeted the C-terminal pseudokinase domain of MLKL to inhibit its membrane translocation and necroptosis [14,44]. Similarly, BLI binding assays found that GW806742X could bind to AjMLKL (S3A and S3B Fig). The dissociation constant (Kd) of GW806742X and AjMLKL is 27.28 μM, so GW806742X was used in subsequent AjMLKL inhibition experiments. Treatment with GW806742X less than 100 μM per sea cucumber did not affect the viability of coelomocytes (Fig 2K). Thus, coelomocytes were treated with 100 μM GW806742X and then infected with 10^8^ CFU/mL AJ01 for another 3 h. We observed a significant reduction in the membrane localization of AjMLKL after GW806742X treatment (S3C and S3D Fig). Meanwhile, GW806742X treatment significantly promoted coelomocyte viability (Fig 2L) and inhibited the cytoplasmic membrane leakage induced by AJ01 infection (Fig 2M) and the proportion of Annexin V+/PI+ lytic coelomocytes (Fig 2N). Consistently with siRNA-mediated knockdown, GW806742X treatment significantly increased the number of intracellular AJ01 survival by 1.55-fold (Fig 2O). These results demonstrated that AjMLKL was involved in AJ01 induced lytic coelomocyte death through its membrane transportation.

### AjMLKL shows membrane lipids binding capacity and cytotoxicity via its N-terminal 4HB domain

**Fig 3:**
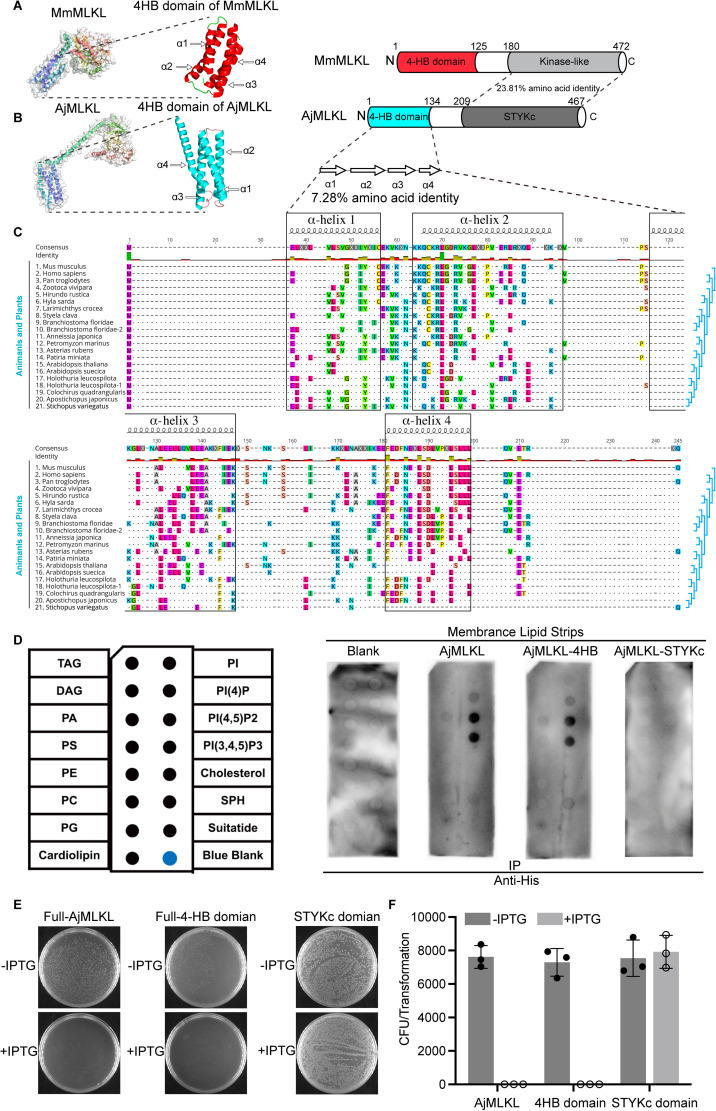
The N-terminal domain of AjMLKL directly binds membrane lipids similar to MLKL four-helix bundle domain. (A) Model (Protein Data Bank accession code 4BTF) and schematic of mouse MLKL, showing the structure of mouse MLKL and Its 4HB domain. (B) Model of AjMLKL and schematic, AlphaFold2 to predict the structure of AjMLKL and showing the N-terminal 4HB domain. Bottom, α-helices are shown as white, right-facing arrows. Helicase and peptidase domain annotations are based on NCBI conserved domains. (C) Amino acid sequence alignment for 4HB domains generated using MUSCLE with representative sequences from vertebrates, plants and invertebrates. Helix boundaries are derived from the predicted structure of mouse MLKL. The top row shows the consensus sequence. Conserved amino acid residues are individually coloured. X and dots indicate hypervariable sites. Cladograms (light blue) display known different species relationships. (D) Membranes displaying lipids were incubated with indicated proteins and binding was assessed by blotting for AjMLKL, AjMLKL-4HB and AjMLKL-STYKc. (E) Representative agar plates showing transformed *E. coli* colonies for AjMLKL, AjMLKL-4HB and AjMLKL-STYKc. (F) bacterial number was counted to test bactericidal activity of AjMLKL (n=3, two-tailed t test) Values are expressed as mean ±S.E.M. (***p<0.001).

During lytic cell death, the lytic nature depends on the phospholipid-binding activity and cytotoxicity of the 4HB domain of MLKL [13]. However, no conserved 4HB domain was predicted from the amino acid sequence of AjMLKL by SMART analysis. To determine the existence 4HB domain in AjMLKL, we used AlphaFold2 to predict the tertiary structure of AjMLKL compared with mouse MLKL protein (PDB: 4BTF). The results showed that the N terminal region of AjMLKL (1-134aa) consisting of 4 α-helix showed high structure similarity with the 4-HB domain of mouse MLKL (Fig 3A and 3B). Sequence analysis of 19 cellular MLKL sequences from animals and plants further revealed residual sequence identities between the N-terminal four α-helical regions of AjMLKL and the 4HB domains of vertebrates and plants (Fig 3C).

To address the phospholipid-binding activity and cytotoxicity functions of AjMLKL 4HB domain, the recombinant AjMLKL (1-467aa), AjMLKL-4HB domain (1-134aa) and STYKc domain (209-467aa) proteins were generated and purified. AjMLKL-4HB domain and AjMLKL proteins showed strong binding with three phospholipids, including phosphatidylinositol 4-phosphate (PI4P), phosphatidylinositol (4,5)-bisphosphate (PIP2) and phosphatidylinositol (3,4,5)-triphosphate (PIP3), while weak binding affinities with the other tested lipids (Fig 3D). The STYKc domain showed no binding activities to any examined lipids (Fig 3D). Meanwhile, AjMLKL-4HB domain and AjMLKL proteins showed high cytotoxicity to *Escherichia coli*, whereas no such function was observed in the recombinant STYKc domain of AjMLKL (Fig 3E and 3F). All these results suggested that the N terminus 4HB domain was responsible the phospholipid-binding activity and cytotoxicity functions of AjMLKL.

### AjMLKL serves as a substrate of active AjCASP-1

**Fig 4:**
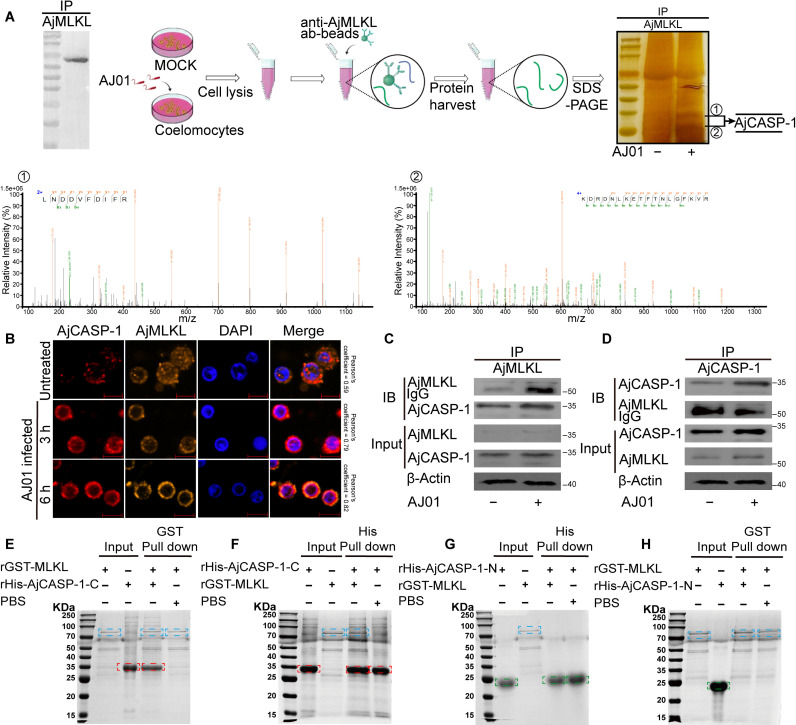
AjMLKL interacts with AjCASP-1 via 4HB and CASc domain. (A) Schematic diagram to detect AjMLKL interactome by precipitation and mass spectrometry. Identification of the protein bound to AjMLKL by mass spectrometry. The protein was identified to be AjCASP-1. (B-D) To define the interaction between AjMLKL and AjCASP-1. AjCASP-1 was found to co-localize with AjMLKL by immunofluorescence (B) (Scale bar, 5 μm.) and Co-IP assays to analyze the interaction between AjMLKL with AjCASP-1 *in vivo* (C and D). (E-H) Different regions of AjCASP-1 and AjMLKL were generated *in vitro*, and their interactions were further validated by using pull-down assays.

To trigger pyroptosis, another type lytic cell death, the activation of GSDMD depends on its cleavage by activated CASP-1 [45]. In our case, AjMLKL also contained a tetrapeptide sequence 14-LVAD-17, which was predicted by PeptideCutter (https://web.expasy.org/peptide_cutter/), and was also specifically recognized and cleaved at Asp17 by inflammatory AjCASP-1 (S4A Fig). More importantly, the size of the smaller AjMLKL product during AJ01 infection was predicted to be identical to AjCASP-1(MH807451) cleaved AjMLKL (Fig 2A). To confirm the AjCASP-1 may act as an executor of AjMLKL cleavage, AjMLKL interacting proteins were identified by IP-MS assay (Fig 4A). By silver staining and IP-MS, we were able to detect two specific bands identical to AjCASP-1 (Fig 4A). Immunofluorescence analysis indicated that AjMLKL could colocalize with AjCASP-1 in coelomocytes, and the signal was enhanced after AJ01 infection (Fig 4B). We then validated the interaction between AjCASP-1 and AjMLKL by Co-IP assay. The results showed a clear interaction between AjCASP-1 and AjMLKL, which was stronger after AJ01 infection (Fig 4C and 4D). Furthermore, we constructed the recombinant proteins AjCASP with truncated C or N terminus to determine the specific interacting domain of AjCASP-1 with AjMLKL by using *in vitro* GST- and His-pull down assays. The results showed that AjMLKL bound to AjCASP-1-CASc (AjCASP-1-C) domain (Fig 4E and 4F), but not to AjCASP-1-N domain (Fig 4G and 4H).

**Fig 5:**
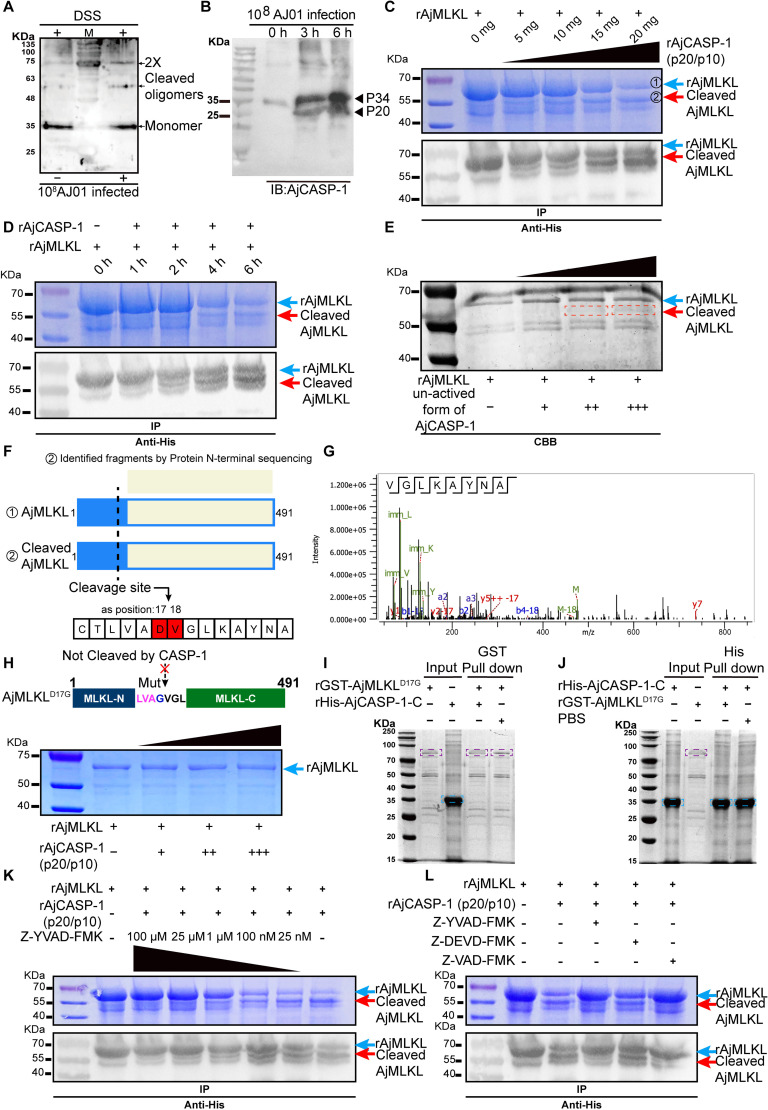
AjMLKL is specifically cleaved by the activated form of AjCASP-1(p20/p10). (A) To detect the active product of AjCASP-1, different AjCASP-1 dimers detected in AJ01 challenged coelomocytes after treatment with a cross-linker disuccinimidyl suberate (DSS). (B) A novel 25 kDa active fragment (P20) of AjCASP-1 was detected after AJ01 infection by Western blot. (C and D) AjMLKL was incubated with either different micrograms of activated form of AjCASP-1(p20/p10) for 3 h (C), or with 10 micrograms of activated form of AjCASP-1(p20/p10) for different hours (D). The samples were then coomassie blue staining and immunoblotting as above. The two AjMLKL fragments are indicated by blue and red arrows, respectively. (E) AjMLKL was incubated with different micrograms of inactivated form of AjCASP-1for 3 h. (F and G) Bands from (C) were analyzed by mass spectrometry to identify AjMLKL cleavage sites. Schematic of AjMLKL coverage by Edman sequencing identified peptides and schematic of AjCASP-1 cleavage site in AjMLKL, AjCASP-1 cleavage of AjMLKL at D17-V18 was monitored by Edman sequencing (F). The N-terminal secondary mass spectra of cleaved AjMLKL are shown in (G). (H) Cartoon diagram of mutant AjMLKL structure and the not cleavage by AjCASP-1 (upper panel) and the influence of the D17G substitution on AjMLKL on its cleaving by AjCASP-1(lower panel). (I and J) Effect of mutation of D17G on AjMLKL by pull down assay on its interaction with AjCASP-1. (K) AjMLKL was incubated with AjCASP-1 in the presence of different concentrations of Z-YVAD-FMK, and the cleavage was determined by coomassie blue staining and immunoblotting immunoblotting as above. (L) AjMLKL was incubated with AjCASP-1 in the presence of different caspase inhibitors, and the cleavage was determined by coomassie blue staining and immunoblotting immunoblotting as above.

In mammalian, the activated CASP-1 is a tetramer consisting of two p20 and two p10 subunits (p20/p10) [46]. In the present study, oligomerization analysis results showed that AjCASP-1 self-cleaved to a dimer at ~60 KDa in response to AJ01 infection (Fig 5A). Western blot analysis showed that the 34 kDa monomer AjCASP-1 was self-cleaved into a 25 kDa protein (p20) in response to AJ01 infection (Fig 5B). Simultaneously, AjCASP-1 activity was significantly upregulated after AJ01 infection (S4B Fig), which could be inhibited by 25 μm of Z-YVAD-FMK (CASP-1-specific inhibitor) without reduce coelomocyte viability in the uninfected coelomocytes (S4C–S4D Fig). All these results suggested that AJ01 infection promoted AjCASP-1 activation to generate the active p20/p10 form.

We then performed *in vitro* cleavage assay to test whether AjMLKL was directly cleaved by the activated form of AjCASP-1. The result showed that the activated form of AjCASP-1 was able to cleave AjMLKL in a dose- and time-dependent manner (Fig 5C and 5D). Instead, full-length AjCASP-1^1-299aa^ had less ability to cleave AjMLKL (Fig 5E). Furthermore, Edman sequencing also indicated that the cleavage site on AjMLKL by activated AjCASP-1 was between Asp17 and Val18 (Fig 5F and 5G). The result was consistent with the predicted 14-LVAD-17 of AjMLKL as the substrate recognition motif of AjCASP-1. To further verify Asp17 of AjMLKL sequence was the key cleavage site for activated AjCASP-1, we constructed a MLKL^D17G^ plasmid in which Asp17 of AjMLKL was replaced by Gly. As a result, rMLKL^D17G^ protein loss the binding ability with AjCAS-1-CASc domain and could not be cleaved by the active AjCASP-1 (Fig 5H–J). In addition, human CASP-1 was also shown to cleave AjMLKL by recognizing the 14-LVAD-17 tetrapeptide sequence on the AjMLKL-4HB domain (S5 Fig). The specificity of AjMLKL cleavage by AjCASP-1 was further confirmed by introducing different CASP inhibitors into the system. The presence Z-YVAD-FMK and Z-VAD-FMK, which could block the proteolytic activity of AjCASP-1, inhibited the production of cleaved AjMLKL (Fig 5K). In contrast, other specific CASP inhibitors could not inhibit AjCASP-1 mediated AjMLKL cleavage (Fig 5L). These results together indicated that AjMLKL was specifically cleaved by the active form of AjCASP-1 after AJ01 infection.

### Cleaved AjMLKL^18-491^ breaks phosphatidylinositol phosphate- and cardiolipin-containing membranes to induce lytic coelomocytes death with large pore sizes

**Fig 6:**
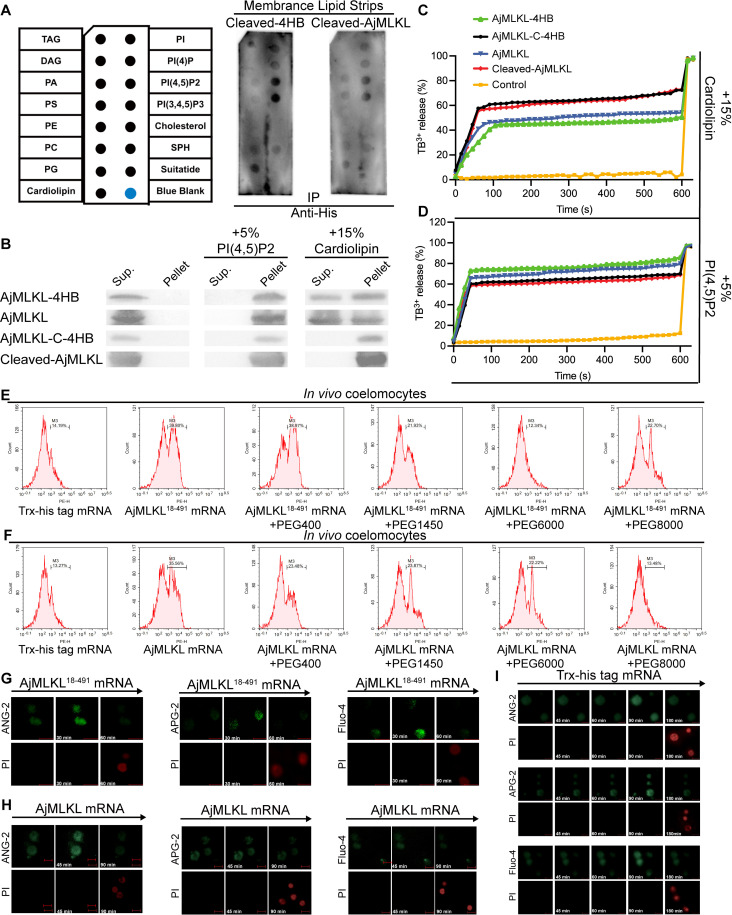
Cleaved AjMLKL^18-491^ breaks phosphatidylinositol phosphate- and cardiolipin-Containing Membranes to induce lytic coelomocytes death with large pore sizes. (A) To search for the molecular mechanism of cleaved AjMLKL membrane association, protein-lipid binding assay was performed with cleaved AjMLKL-4HB domain and cleaved AjMLKL. (B) AjMLKL, AjMLKL-4HB, Cleaved-AjMLKL and Cleaved-AjMLKL-4HB domain binding to PC–PE liposomes containing additional indicated phospholipids (molar proportion of added lipid indicated) was analysed by SDS–PAGE and AjMLKL immunoblot. (C-D) Time course of liposome leakage was monitored as described in the Experimental Procedures. Asterisks indicated the time points when Triton X-100 was added to achieve complete release of Tb^3+^. Comparison of AjMLKL, AjMLKL-4HB, Cleaved-AjMLKL and Cleaved-AjMLKL-4HB domain in liposome leakage assay. Liposomes containing cardiolipin (CL) (C) or PIP2 (D) were used as indicated. Curves in different colors represent purified recombinant AjMLKL-4HB, AjMLKL, Cleaved-AjMLKL-4HB domain and Cleaved-AjMLKL. (E and F) Uncleaved MLKL or cleaved MLKL overexpressed coelomocytes were treated with AJ01 for 3 h in PBS (CTRL) or PBS containing 30 mM PEG400, PEG1450, PEG6000 or PEG8000. Cells were stained by PI and analyzed under Flow cytometry. (G-I) Intracellular ion concentration were monitored in uncleaved MLKL (G) or cleaved MLKL (H) overexpressed coelomocytes treated with AJ01. Sodium indicator (ANG-2), Calcium indicator (Fluo-4) and Potassium indicator (APG-2) were used together with PI. Trx-His-Tag mRNA overexpressing coelomocytes were treated with AJ01 as a control (I).

To validate whether cleaved AjMLKL^18-491^ facilitated lytic coelomocyte lytic death, we generated recombinant AjMLKL^18-491^ and the 4HB domain (4HB^18-134^ domain) for lipid binding assay. AjMLKL^18-491^ displayed additional high binding capacities to cardiolipin, phosphatidylinositol (PI), PA and PS compared to un-cleaved AjMLKL, despite a similar binding capacity to PI4P, PIP2 and PIP3 (Fig 6A). While the 4HB^18-134^ domain of AjMLKL had a similar lipid binding activity with AjMLKL^18-491^ (Fig 6A). Cleaved AjMLKL^18-491^ and 4HB^18-134^ domain also exhibited stronger binding (Fig 6B) and leakage capacity for liposomes composed of 15% cardiolipin compared to those of un-cleaved AjMLKL and 4HB domain (Fig 6C). While there were no significant differences for liposomes composed of 5% PI(4,5)P2 (Fig 6B and 6D).In addition, recombinant AjMLKL^18-491^ and 4HB^18-134^ domain also showed toxic to *E. coli* (S6 Fig).

Lytic cell death could forms different sized pore in membrane, in which the small permeable pores of necroptosis was mediated by phosphorylated MLKL [4,47], while the large pores of pyroptosis is mediated by cleaved GSDMD-N [4,48,50]. Polyethylene glycol (PEG) of certain size is widely used as the inhibitor for the detection pore formation [51]. We found that coelomocyte lytic death induced by overexpression of cleaved AjMLKL^18-491^ could be significantly inhibited by PEG1450, PEG6000, and PEG8000, but not PEG400 (Fig 6E), In the un-cleaved AjMLKL overexpressed group, PEG400, PEG1450, PEG6000, PEG8000 all significantly inhibited lytic coelomocytes death compared to the control group (Fig 6F). This result implied that cleaved AjMLKL^18-491^ formed large pore sizes in the cell membrane similar to pyroptosis, whereas un-cleaved AjMLKL formed small pore sizes similar to necroptosis.

The eventual cell rupture of lytic cell death was produced in two different ways, one maybe caused by the influx of selective ion pore-driven water formed by MLKL in necroptosis, however, exactly how MLKL was involved in this process is currently full of controversy [36,37,52,53]. The other by the influx of non-selective ion pore-driven water formed by GSDMD-N in pyroptosis [36,54]. To address the change of intracellular ion homeostasis during AjMLKL^18-491^-mediated lytic coelomocyte lytic death, sodium indicator ANG-2, potassium indicator APG-2, and calcium indicator Fluo-4 were used according to previously reported [55–57]. When overexpressed cleaved MLKL^18-491^ in coelomocytes, the intensity of ANG-2 first increased and then abruptly decreased when the plasma membrane was ruptured, as reflected in PI uptake (Fig 6G), compared with the Trx-his tag mRNA overexpression group (Fig 6I). Similar results were observed when staining with APG-2 and Fluo-4 (Fig 6G), which suggested that cleaved AjMLKL^18-491^ forms non-selective ionic pores in the cell membrane. In contrast, un-cleaved AjMLKL showed selectivity for ANG-2 (Fig 6H), but not APG-2 or Fluo-4 (Fig 6H), which suggested that un-cleaved AjMLKL forms non-selective ionic pores in the cell membrane. Flow cytometry analysis also confirmed these results (S7 Fig).

### Cleaved AjMLKL directly kills AJ01

**Fig 7:**
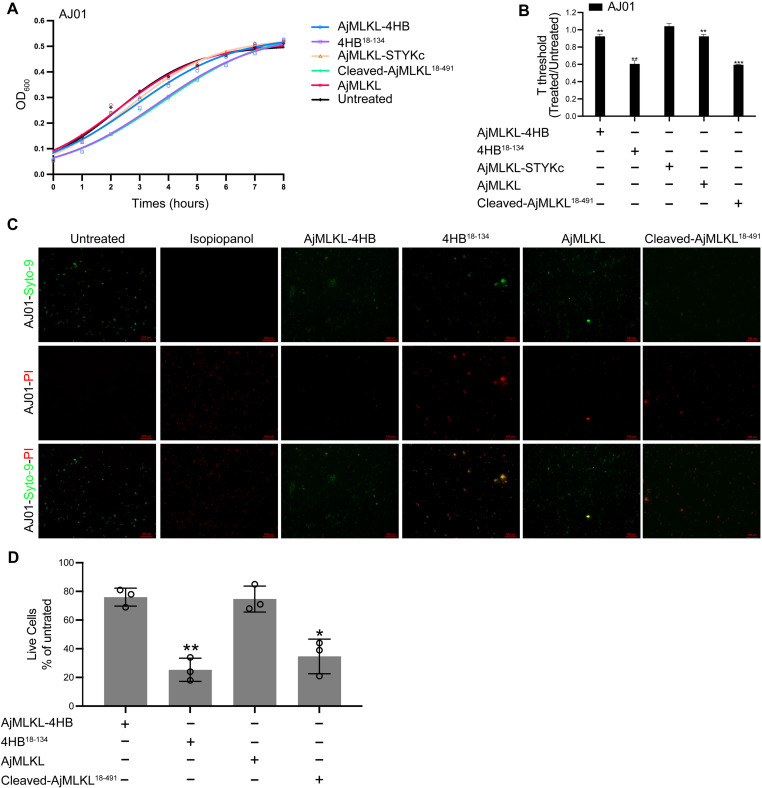
Cleaved AjMLKL directly kills AJ01. (A and B) AJ01 were untreated or treated with recombinant AjMLKL-4HB domain, AjMLKL, Cleaved-AjMLKL-4HB domain or Cleaved-AjMLKL^18-491^ (200 nM or indicated concentrations) for 30 min before samples were collected and bacterial growth was assessed by monitoring turbidity by optical density (representative experiments, left) (A). The time to reach OD_600_ of 0.05 above background, which is a quantitative measure of the lag in detectable growth because of fewer viable bacteria, was defined as T_threshold_ (right). The right graph shows the mean ± s.d. of three technical replicates (B). (C and D) AJ01 viability assays, Bacterial viability after 20 min incubation with indicated, proteins (200 nM) or isopropanol. Syto-9 enters live and dead bacteria, PI only enters dead bacteria (representative images, left; percent live cells, right). Data shown are representative of results of three independent experiments. Statistical differences are relative to untreated samples; * ****P**** < 0.05, **P < 0.01 (two-tailed t-test). Scale bars, 5 μm.

The cleavage of AjMLKL by activated AjCASP-1 mediates lytic coelomocytes death in a manner similar to the activation of mammalian GSDMD mediated pyroptosis, and the cleaved GSDMD N-terminus that can not only mediate pyroptosis but can also killed intracellular bacteria directly [49]. To clarify whether the cleaved AjMLKL^18-491^ could be served as antibacterial protein as the cleaved GSDMD-N terminus. We measured the growth of AJ01 after incubation with nanomolar concentrations of recombinant AjMLKL-4HB domain, AjMLKL, 4HB^18-134^ domain or cleaved AjMLKL^18-491^ (Fig 7A and 7B). Cleaved AjMLKL^18-491^ and 4HB^18-134^ domain exhibited stronger anti-bacterial activity against AJ01 compared to the un-cleaved AjMLKL as well as its 4HB domain (Fig 7A and 7B). To further clarify whether AjMLKL inhibited the growth of AJ01 through a direct bactericidal effect, the bacterial LIVE/ DEAD assay was used. The results revealed that cleaved AjMLKL^18-491^ killed approximately 75% of AJ01 (Fig 7C and 7D) through its 4HB^18-134^ domain, whereas un-cleaved AjMLKL and AjMLKL-4HB domains killed only approximately 22% of AJ01 (Fig 7C and 7D). The above results clarified the cleaved AjMLKL^18-491^ not only induced lytic coelomocytes death, but also exhibited a very extreme direct killing AJ01.

### Activation of AjCASP-1 is dependent on the assembly of AjNLRC4 inflammasome-like complex

**Fig 8:**
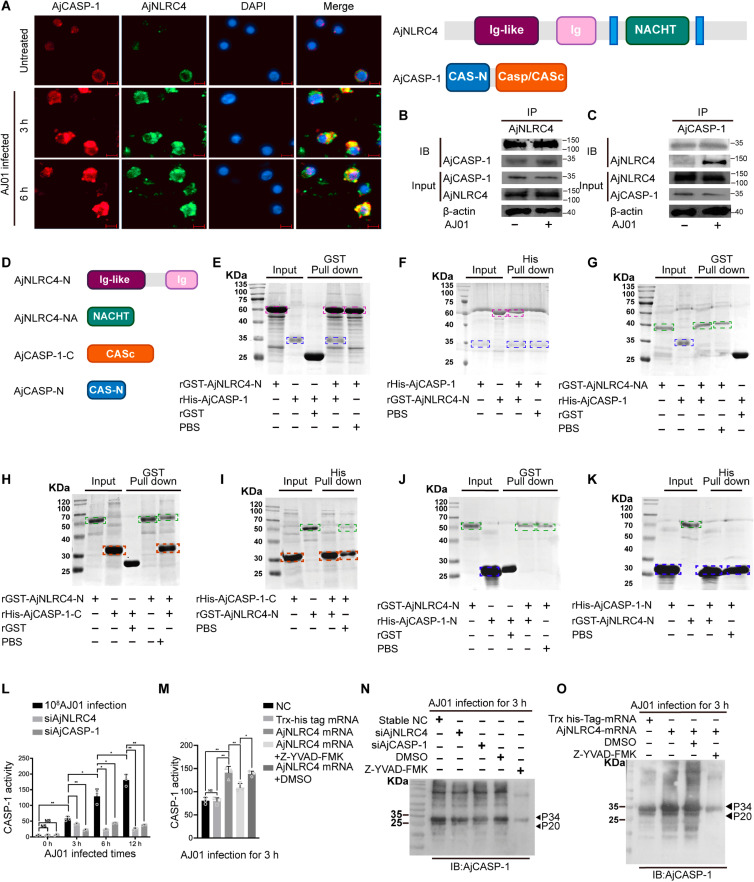
Activation of AjCASP-1 is dependent on the assembly of AjNLRC4/AjCASP-1 inflammasome. (A) Coelomocyte AjNLRC4 and AjCASP-1 were found to be colocalized in cytoplasm by immunocytochemistry analysis, and the signal intensity was enhanced after AJ01 infection. (Scale bar, 5 μm.). (B and C) The coelomocytes were harvested after infection with 10^8^ CFU/ml AJ01 at different time points (0 and 3 h). The interaction between AjNLRC4 and AjCASP-1 was further determined by Co-IP and Western blotting. (D) Different domain recombinant proteins of AjNLRC4 and AjCASP-1 were generated in vitro. (E-K) The interaction between AjNLRC4 and AjCASP-1 were further validated by using pull-down assays. The product was isolated by SDS-PAGE and detected by Coomassie blue staining. Dotted boxes of different colors represent recombinant proteins of different domains of AjNLRC4 and AjCASP-1 (L) To further validate AjNLRC4’s role in activating AjCASP-1, coelomocyte CASP-1 activity was detected with specific Ac-YVAD-pNA fluorescent substrates after the silencing of AjCASP-1 or AjNLRC4 and challenge with 10^8^ CFU/ml AJ01 infection for 0, 3, 6, and 12 h. (M) To further elucidate AjCASP-1 involved in AJ01-mediated **lytic coelomocytes death**, commercial CASP-1 specific inhibitor Z-YVAD-FMK did not affect coelomocyte viability at 25 μM. (N) CASP-1 activity was assayed by treatment with 25 μM Z-YVAD-FMK or DMSO (control) for 3 h and then challenged with 10^8^ CFU/ml AJ01 for 3 h. (O) Coelomocyte CASP-1 activity was assayed under AjNLRC4 overexpression conditions. Consistently, the overexpression of AjNLRC4 promoted the production of P20, the addition of Z-YVAD-FMK inhibited the AjNLRC4 on AjCASP-1 self-cleavage. All data are plotted as mean ± SEM, asterisks indicate significant differences: *p < 0.05, **p < 0.01, ***p < 0.001.

In higher mammals CASP-1 activation is dependent on the assembly of NLR receptors with CASP-1 to form inflammasomes via CARD domain [58–60]. CASP-1 in *A. japonicus* lacked the typical CARD domain and had a conserved CASc domain (S8A–D Fig). NLRC4 in sea cucumbers (AjNLRC4, MN607598) lacks the typical CARD and LRR domains and is instead composed of Ig domain and NACHT domains (S8E–H Fig). Under AJ01 infection, NLRC4 is internalized from the cell membrane into the cytoplasm [61]. To investigate whether atypical AjNLRC4 and AjCASP-1 could form an inflammasome-like complex to activate AjCASP-1, the interaction between AjNLRC4 and AjCASP-1 was analyzed by immunofluorescence, Co-IP, and pull-down assays. Immunofluorescence analysis indicated that AjNLRC4 could colocalize with AjCASP-1, and the complex signal was significantly enhanced after 10^8^ CFU/mL AJ01 infection (Fig 8A), which was consistent with increased AjCASP-1-interactive AjNLRC4 intensity by AjCASP-1 antibody as a bait (Fig 8B) and increased AjCASP-1 intensity by AjNLRC4 antibody for Co-IP analysis (Fig 8C). Furthermore, we constructed the recombinant proteins of truncated AjNLRC4 and AjCASP-1 to determine the specific interaction domain between AjNLRC4 and AjCASP-1 by using *in vitro* GST- and His-pull down assays (Fig 8D). The results showed that AjCASP-1 interacted with AjNLRC4-Ig-like and Ig-like domain (Fig 8E and 8F), but had no interaction with AjNLRC4-NACHT domain (AjNLRC4-NA) (Fig 8G). AjNLRC4-Ig-like and Ig domain (AjNLRC4-N) bound to AjCASP-1-CASc domain (AjCASP-1-C) (Fig 8H and 8I), but not to AjCASP-1-N (Fig 8J and 8K). These results suggested that AjNLRC4 bound to the CASc domain of AjCASP-1 via its Ig-like and Ig domain to form an inflammasome-like complex in response to AJ01 infection.

To clarify the regulation of AjCASP-1 activity by AjNLRC4, AjCASP-1 activity was assayed after silencing of AjNLRC4. We found that AjNLRC4 interference significantly inhibited the cleavage of Ac-YVAD-pNA (Fig 8L) *in vivo*. To further determine whether AjNLRC4-mediated lytic coelomocyte death depending on the activity of AjCASP-1, AjCASP-1 activity was assayed in AjNLRC4 overexpression and Z-YVAD-FMK treatment conditions. The results showed that AjNLRC4 -mediated cleavage of Ac-YVAD-pNA could be blocked by Z-YVAD-FMK treatment (Fig 8M). Consistently, interference of AjNLRC4 also significantly decreased p20 protein production (Fig 8N), and the production of AjCASP-1 self-cleavage product p20 by AjNLRC4 overexpression could be inhibited by addition of Z-YVAD-FMK (Fig 8O).

### AjNLRC4 modulates AJ01-induced lytic coelomocyte death depending on AjCASP-1

**Fig 9:**
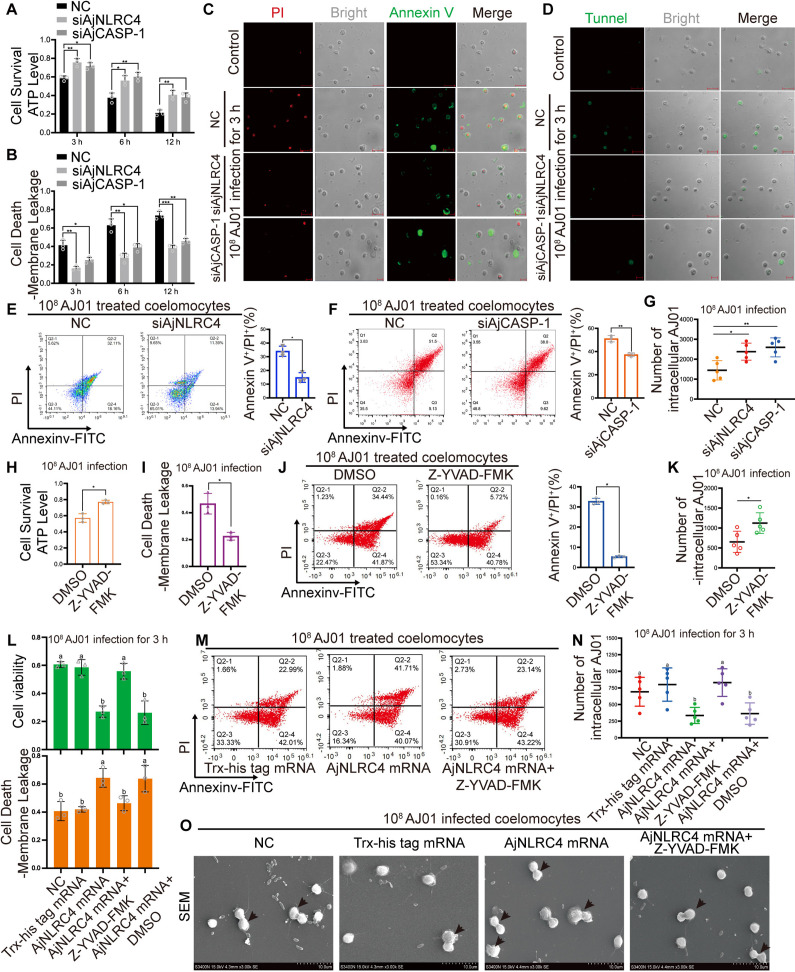
AjNLRC4 modulates AJ01-induced lytic coelomocyte death depending on AjCASP-1. (A-G) Sea cucumbers were transfected with specific AjNLRC4 siRNA or AjCASP-1 siRNA for 24 h. The nontargeting siRNA (NC) was transfected as the control group. Then, 10^8^ CFU/mL AJ01 at final concentration was added to the experimental and NC groups for another 3, 6, and 12 h. The collected coelomocytes were used to determine coelomocyte viability (A), and coelomocytes plasma membrane disruption (B). (C and D) Coelomocytes from the control group (untreated sea cucumber), NC group, and experimental group (3 h) were used for annexin V and PI double staining (C) and tunnel staining assays (D), detected by fluorescence microscopy. (E and F) The proportion of Annexin V+/PI+ necrotic coelomocytes were texted by Flow Cytometry. (G) The intracellular AJ01 survival was determined by plate counts. (H-K) To further elucidate the regulation of CASP-1 activity by AjCASP-1, commercial CASP-1 specific inhibitor Z-YVAD-FMK did not affect coelomocyte viability at 25 μM. Sea cucumbers were injected with Z-YVAD-FMK inhibitor for 3 h, followed by 10^8^ CFU/mL Aj01 infection for another 3 h to determine coelomocyte viability (H) and coelomocytes plasma membrane disruption (I). The proportion of Annexin V+/PI+ lytic coelomocytes death (J) and intracellular AJ01 survival (K) were also assayed in the AJ01 + Z-YVAD-FMK and AJ01 + DMSO groups. (L-O) To confirm whether AjCASP-1-mediate **lytic coelomocytes death** is associated with AjNLRC4, AjNLRC4 mRNA was overexpressed by transfection of AjNLRC4 mRNA, and then Z-YVAD-FMK was injected into the overexpression group for 3 h, followed by 10^8^ CFU/mL AJ01 infection for another 3 h. The transfection of Trx-His tag mRNA served as the control group for AjNLRC4 overexpression group. DMSO served as the control for Z-YVAD-FMK treatment. Coelomocyte viability (L), coelomocytes plasma membrane disruption (L), and the number of intracellular AJ01 survival (N) were assayed in different groups. The percentage of annexin V+/PI+ coelomocytes were assayed by flow cytometry (M). FEG-SEM was used in observing morphological changes in coelomocytes (Q), (scale bar, 10 μm). All data are plotted as mean ± SEM, asterisks indicate significant differences: *p < 0.05, **p < 0.01, ***p < 0.001.

To validate the role of atypical AjNLRC4 and AjCASP-1 in AJ01 infection-induced lytic coelomocyte death in sea cucumber, lytic coelomocyte death was assayed after the silencing of AjNLRC4 or AjCASP-1 by specific small-interfering RNA (siRNA) (S1 Table and S9A and S9B Fig). The results revealed that compared with the nontarget siRNA transfection group (NC), knock down AjNLRC4 or AjCASP-1 significantly increased the coelomocyte viability (Fig 9A) and decreased the leakage of cell membrane and the number as well as the proportion of Annexin V+/PI+ lytic coelomocytes induced by 10^8^ CFU/mL AJ01 (Fig 9B–F). Consistently, the number of intracellular AJ01 survival (Fig 9G) were significantly increased in the experimental group. Z-YVAD-FMK treatment increased coelomocyte viability to 77.02 ± 2.26% (*p* < 0.05, Fig 9H), whereas cell membrane leakage significantly decreased to 22.55 ± 2.14% (*p* < 0.05, Fig 9I) relative to the those in the AJ01+DMSO group (57.15 ± 5.29% and 46.73 ± 7.68%, respectively). The proportion of Annexin V+/PI+ necrotic coelomocytes (Fig 9J) and intracellular AJ01 level significantly decreased in the AJ01 + Z-YVAD-FMK group compared with the AJ01 + DMSO group (Fig 9K). These results indicated that AjNLRC4 and AjCASP-1 were involved in AJ01-induced lytic coelomocyte death in sea cucumber.

To further confirm whether AjCASP-1-mediated lytic coelomocyte death associated with AjNLRC4, we overexpressed the mRNA of AjNLRC4 by mRNA transfection for 24 h. The results found that AjNLRC4 protein level increased by 1.86-fold (S10A Fig) compared with that in the AJ01 + Trx-His tag mRNA group. In this condition, AjNLRC4 overexpression decreased coelomocyte viability to 0.64-fold (Fig 9L) and increased cell membrane leakage to 1.53-fold (*p* < 0.01, Fig 9L) compared with those in the AJ01 + Trx-His tag mRNA group. AjNLRC4 overexpression increased the proportion of Annexin V+/PI+ necrotic coelomocytes to 1.52-fold (*p* < 0.05) and decreased intracellular AJ01 level to 0.41-fold (*p* < 0.05, Fig 9M and 9N). Coelomocytes with typical lytic cell death morphology were observed in the AJ01 + AjNLRC4 mRNA group (Fig 9O). AjNLRC4-induced lytic coelomocyte death was blocked by of Z-YVAD-FMK administration (Fig 9O). Z-YVAD-FMK treatment upregulated coelomocyte viability to 55.81 ± 5.61% (*p* < 0.01, Fig 9L) and reduced cell membrane leakage to 46.35 ± 5.29% (*p* < 0.05, Fig 9L) compared with those in the AJ01+ AjNLRC4 mRNA group (27.06 ± 4.01% and 64.45 ± 6.67% respectively). Similarly, Z-YVAD-FMK treatment downregulated AjNLRC4-induced Annexin V+/PI+ necrotic coelomocytes from 39.91 ± 4.28% to 29.58 ± 4.01% (*p* < 0.05, Fig 9M), and intracellular AJ01 level increased to 2.28-fold (*p* < 0.01, Fig 9N).

### AjNLRC4/AjCASP-1 induces AjMLKL cleavage and membrane migration to mediate lytic coelomocyte death

**Fig 10:**
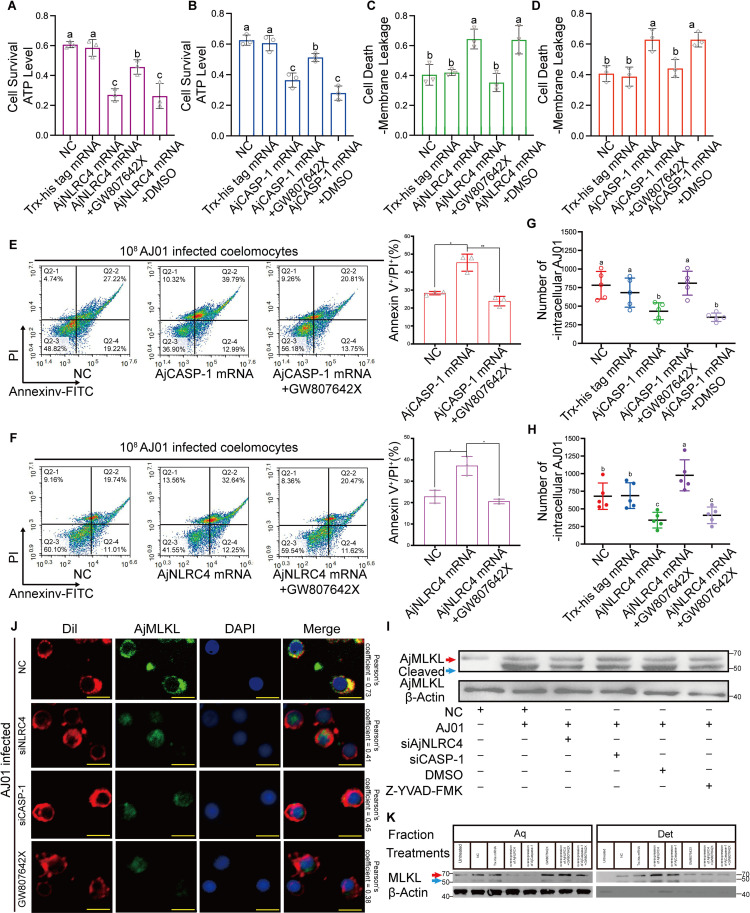
AjNLRC4/AjCASP-1 induces cleaving of AjMLKL and mediates lytic coelomocytes death by regulating the migration of cleaved AjMLKL to the cell membrane. (A-H) To confirm whether AjMLKL-mediated lytic coelomocytes death was associated with AjNLRC4 or AjCASP-1, the mRNA of AjNLRC4 or AjCASP-1, and then GW806724X was injected into the overexpression group for 3 h, followed by 10^8^CFU/mL AJ01 for another 3 h. Coelomocyte viability (A and B) and coelomocytes plasma membrane disruption (C and D) were assayed. The percentage of Annexin V+/PI+ coelomocytes (E and F) and intracellular AJ01 survival were determined under the same condition (G and H). (I) Interference with AjNLRC4 or AjCASP-1 and treatment with CASP-1 inhibitor Z-YVAD-FMK inhibited MLKL cleavage. (J) Interference by AjNLRC4 or AjCASP-1 or GW806724X treatment significantly inhibited the migration of AjMLKL and its cleavage products from the cytoplasm to the cell membrane, (scale bar, 5 μm). (K) Interference with AjNLRC4 or AjCASP-1 and treatment with the MLKL inhibitor GW806724X both blocked translocation of cleaved MLKL to the membrane fraction. The cells were harvested and then separated into the aqueous phase and detergent phase, performed as described in (Fig 2D). The samples were analyzed by western blotting using antibodies as indicated. All data are plotted as mean ± SEM, asterisks indicate significant differences: *p < 0.05, **p < 0.01, ***p < 0.001.

We then investigated whether AjMLKL-mediated lytic coelomocyte death associated with AjNLRC4 or AjCASP-1, The results showed that GW806724X treatment could block AjNLRC4 or AjCASP-1 overexpression (S10B Fig) induced the decreased coelomocyte viability (Fig 10A and 10B) and intracellular AJ01 survival (Fig 10G and 10H), and also reverse the increased cell membrane leakage (Fig 10C and 10D) and the proportion of Annexin V+/PI+ necrotic coelomocytes (Fig 10E and 10F).

To further confirm AjNLRC4/AjCASP-1 induced lytic coelomocyte death depending on AjMLKL cleavage and migration, AjMLKL cleavage and migration were analyzed after AjNLRC4/AjCASP-1 abnormal conditions. The results revealed that interference AjNLRC4/AjCASP-1 significantly inhibited the cleavage of AjMLKL (Fig 10I) and the migration of AjMLKL to the cell membrane (Fig 10J). Overexpression of AjNLRC4/AjCASP-1 promote the migration of cleaved AjMLKL^18-491^ towards the cell membrane, which could be blocked by the GW806724X administration (Fig 10K). All these results suggest that AjNLRC4/AjCASP-1 mediates lytic coelomocyte death by regulating AjMLKL cleaving and promoting its migration to the cell membrane.

## Discussion

**Fig 11:**
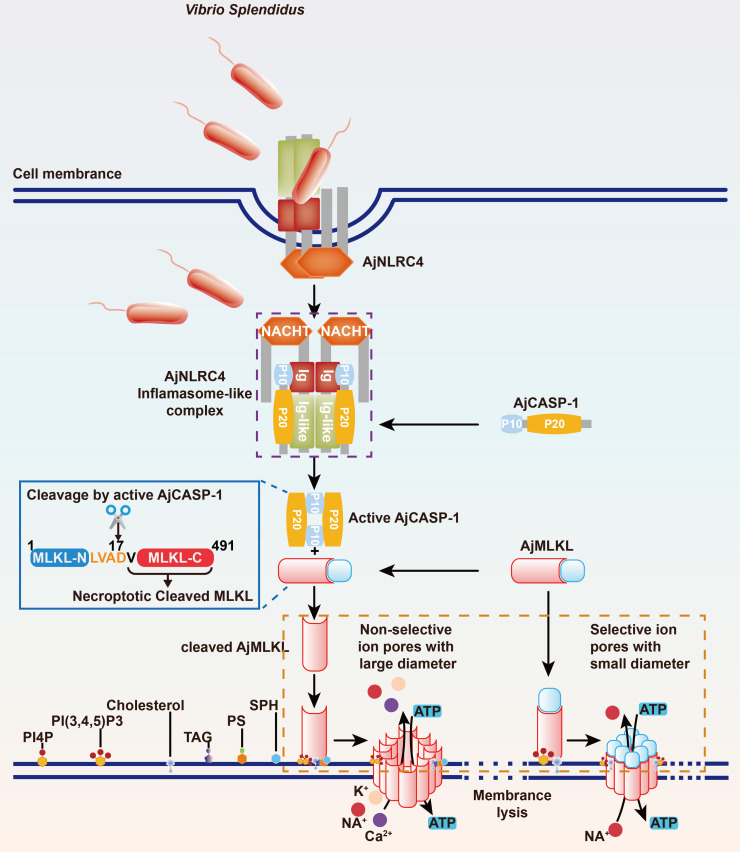
A schematic diagram of atypical NLRC4-CASP-1-MLKL axis-mediated lytic coelomocytes death that is not dependent on MLKL phosphorylation. AJ01 was identified by AjNLRC4 to promote AjNLRC4 dimer formation and internalized into cytoplasm. The interlined AjNLRC4 without CARD domain interacted with the C-terminal of AjCASP-1 through its Ig domain, which also lacked the CARD domain. The interaction further promoted AjCASP-1 activation and generated the active p20/p10 form, which subsequently specifically cleaves AjMLKL at Asp17 in the 14-LESD-17 tetrapeptide. Then, the cleaved AjMLKL is transported to the membrane on the one hand by binding to lipids, forming non-selective ion channels and inducing lytic coelomocytes. On the other hand, it also directly kills AJ01.

MLKL phosphorylation-mediated necroptosis has been extensively studied in higher mammals mainly in humans and mice to combat with pathogen infection [30]. However, MLKL phosphorylation was not detected in AJ01 infected sea cucumber coelomocytes, suggesting a phosphorylation-independent manner might be existed. In the current study, we found a novel CARD domain-independent assembly mode of inflammasome-like complex AjNLRC4/AjCASP-1 and further confirmed the complex activated AjMLKL-mediated lytic coelomocyte death via specific cleaving AjMLKL, rather than relying on the classical MLKL phosphorylation pathway (Fig 11). AjNLRC4 without CARD domain interacted with the CASc domain of AjCASP-1 through its Ig domain. The interaction further promoted AjCASP-1 activation and generated the active p20/p10 form, which subsequently specifically cleaved AjMLKL at ASP17 in the 14-LESD-17 tetrapeptide. Then, cleaved AjMLKL^18-491^ showed higher binding activities to membrane lipid compared to the complete AjMLKL and was further translocated to the membrane via its un-conserved 4HB domain, and finally formed a large non-selective ionic coelomocyte pore to induce lytic coelomocyte death. The cleaved AjMLKL^18-491^ also displayed directly AJ01 elimination activity similar to the cleaved GSDMD, which induced lytic cell death of pyroptosis in a wide range of animals (Fig 11). This was the first report MLKL mediated lytic coelomocyte death via selectively cleavage not phosphorylation, which substantially enhanced our understanding of the ancestral innate immune repertoire on lytic cell death. The results also implied that pyroptosis and necroptosis might be combined in some lower animals without GSDMs.

Evolutionarily, MLKL and RIPK3 appeared early, namely after the bifurcation of Protostomia and Deuterostomia. MLKL gene seemed to have appeared after the bifurcation between Protostomia (Annelida, Mollusca, Arthropoda, and Nematoda) and Deuterostomia (Echinodermata, Hemichordata, and Chordata) as all investigated lineages from the latter and none of the first express MLKL. At present, homologs of MLKL have been found in plants [62], and the 4HB domain, which is important for MLKL to perform its lytic function, has been found in fungi and noroviruses[13,63]. In addition, The MLKL homologs in the above species are poorly conserved with AjMLKL, and there are also obvious differences between MLKL in different species, suggesting that the mechanism of action of MLKL in invertebrates may be quite different. In contrast, the RIPK3 gene only appeared in the Craniata clade, and not in Cephalochordata and Urochordata [30]. Another interesting finding was the multiple replication of MLKL in RIPK3-deficient species such as the continental clawed frog, amphioxus, acorn worm (*Saccoglossus kowalevskii*) and sea urchin (*Strongylocentrotus purpuratus*) [30]. These findings implied that in these lower invertebrates, strictly lytic cell death could not be initiated from the classical kinase-regulated RIPK3-MLKL- necroptotic axis presented in human and mouse cells. These point further supported by the fact that plant *Arabidopsis thaliana* overexpressing AtMLKL, which mimics the phosphorylation site S393D, did not induce cell death in *Arabidopsis thaliana* [62]. In the classical RIPK3-mediated MLKL phosphorylation regulatory pathway, the formation of a stable complex between RIPK3 and MLKL was necessary, in which autophosphorylation at residue S227 in the kinase domain of RIPK3 was required. Mutation of this site caused RIPK3 to lose its binding ability to MLKL and thus failed to induce MLKL phosphorylation [64]. In our work, the S227 site was replaced by N249 in AjRIPK3 (PIK48672.1), which might be the real reason why AjMLKL could not interact with AjRIPK3 and not phosphorylated by AjRIPK3 in response to AJ01 infection despite the coexistence of AjRIPK3. More importantly, Tanzer et al 2016 [21] found that full-length MLKL constructs bearing mutations mimicking RIPK3-mediated phosphorylation, T357E/S358E (TSEE) hMLKL, and S345D mMLKL, did not induce cell death in wild-type or MLKL-/-MDF, HT29 or U937 cells [21]. In contrast, the murine S345D mMLKL mutant caused U937 cell death. All these results supported that MLKL-mediated lytic cell death might not be necessarily caused by phosphorylation alone, and other activation signals and interactions might also be present.

Here, we firstly confirmed that AjMLKL cleavage was involved in lytic coelomocyte death, which served as the substrate of active AjCASP-1. In general, CASP-1 was activated dependent on the formation of inflammasome via NLR oligomerization, which was a universal mechanism in promoting CASP-1 dimerization and auto-processing [46,65]. In our previous studies, AjNLRC4 lacking the CARD domain also formed dimers through its Ig domain that internalized into the cytoplasm upon recognition of pathogens [61]. In this work, we further validated that the AjNLRC4 dimer could recruit and activate CASP-1 like higher vertebrates. However, the mechanism by which AjNLRC4 recruiting CASP-1 differed from the homotypic interaction of higher vertebrates through the CARD domain. AjNLRC4 combined AjCASP-1 through its Ig domain because the Ig domain of AjNLRC4 contained a conservative CASP-1 recognition site (177-LRTD-180). Inflammatory CASP usually recognized and bound proteins with a tetrapeptide motif (LXXD), in which aspartic acid residues were essential for protein–protein interaction [66–69]. Interestingly, no specific recognition motif of other CASP was found in the AjNLRC4 sequence, which indicated that AjNLRC4 specifically bound AjCASP-1. More importantly, molecular docking predictions showed that the p10 region of the CASc domain of AjCASP-1 was the key region for the interaction between AjCASP-1 and AjNLRC4-Ig domain. Similarly, human CASP-1/4/11 bound GSDMD primarily through its p10 domain [70]. Those above analysis implied that the interaction mode of AjNLRC4 and AjCASP-1 may be similar to human CASP-1/4/11 bound GSDMD. After the assembly of AjNLRC4 and AjCASP-1 completed, activated AjCASP-1 was automatically hydrolyzed to the p20 product and formed a p20/p10 dimer. It was attributed to the fact that AjCASP-1 contained a highly conserved CASc structural domain and a conserved caspase active site, QACRG (Gln-Ala-Cys-Arg-Gly), which was conserved in human CASP-1 [69,71]. This suggested that AjCASP-1 had similar substrate binding and catalytic activity to HsCASP-1. Finally, AjCASP-1 was activated on AjNLRC4-inflammasome-like complex, which was similar to conserved signal transduction to promote CASP-1 dimerization and automatic processing in vertebrates [69]. This finding was supported by a recent study demonstrating that active CASP-1 is a p20/p10 dimer [69]. In GSDM-deficient sea cucmber, AjMLKL replaces GSDM to achieve activation in a pathway similar to the canonical inflammasome in mammals. This shows a unique regulatory relationship between MLKL and CASP in sea cucumbers. However, the activation of GSDM in mammals is also achieved by an activation pathway called noncanonical inflammasome [72]. So whether there is an activation pathway similar to the noncanonical inflammasome in sea cucumbers to achieve MLKL activation deserves further in-depth exploration in the future.

In mammals, the activated form of CASP-1 cleaved GSDMD to mediate pyroptosis [73]. However, no members of the GSDMs family of key executive proteins of pyroptosis were found in sea cucumber genome, although GSDMs were widespread in molluscs including scallops, oysters, owl limpet, and blood shell [74]. Surprisingly, necroptosis executor AjMLKL could be cleaved by activated AjCASP-1 similar to GSDMs activation in other species. It had been reported that inflammatory CASPs typically recognize a tetrapeptide motif (LXXD) in the substrate and cleaved after aspartate [67,68]. Just like CASP-1/4/5/11 cleaved MmGSDMD at Asp276 in the 273-LLSD-276 tetrapeptide [45], as well as a recently reported CASP-4 cleaved pro-IL-18 at Asp36 in the 33-LESD-36 tetrapeptide [75]. Similarly, activated form of AjCASP-1 cleaved AjMLKL at Asp17 in the 14-LVAD-17 tetrapeptide. The cleavage activity was dependent on the conserved CASP-1 recognition tetrapeptide motif (14-LVAD-17) in AjMLKL, which fitted well with the substrate recognition motif of inflammatory CASP. Compared to human or mouse MLKL sequences, there was no apoptotic CASP recognition site (DXXD) on AjMLKL, and no inflammatory CASP recognition motifs were present in human, mouse or plant MLKL sequences, suggesting that cleavage of AjMLKL by inflammatory AjCASP-1 might be unique to sea cucumber. lytic coelomocyte death mediated by AjMLKL as a substrate for AjCASP-1 may well complement the loss of pyroptosis caused by the absence of GSDMs in sea cucumber. Because pyroptosis and necroptosis are belonging to lytic cell death, both result in similar features such as cell swelling, rupture of the cell membrane and release of cytoplasmic contents [76], and functional similarities between GSDMs and MLKL. MLKL and GSDMD could bind with negatively charged phosphatidylinositol phosphate (PIP) and promote its redistribution to the plasma membrane [36,37]. In our study cleaved AjMLKL^18-491^ exhibited similar lipid binding capacity to the cleaved GSDMD-N. This was because the lipid-binding profile of MLKL was dependent on its 4HB domain. In human MLKL, a patch of positively charged amino acids on the surface of the 4HB domain bound to PIPs and facilitated the recruitment of MLKL to the plasma membrane [39]. The 4HB domain of cleaved AjMLKL contained positively charged amino acid spots, which allowed cleaved AjMLKL to bind with the plasma membrane. The binding of the cleaved AjMLKL to the plasma membrane eventually induced cell membrane pore-forming damage through its 4HB domain, as this domain had been shown to have direct pore-forming or membrane-disrupting capacity [36,38]. And that cleaved AjMLKL^18-491^-induced lytic coelomocytes death could be inhibited by PEG1450-8000 but not counteracted by PEG400, implying that necrotic cell death regulated by cleaved AjMLKL^18-491^ formed pore channels of 1.1 to 2.4 nm in the cell membrane. This result is consistent with the size of the pores formed in the cell membrane by the pyroptosis mediated by GSDMD-N [48–50]. Changes of cell volume often accompany cell death and are typically driven by ion-involved osmotic pressure [77]. The pores formed by the cleaved AjMLKL^18-491^ were consistent with the non-selective pores formed by GSDMD-N and dissipate natural ionic gradients [4]. This implies the observation of cell swelling in AJ01 pre-infection necrotic cell death, which may be caused by water influx driven by intracellular non-ionic osmolytes [4]. However, it is worth noting that there is currently great controversy over the theory of how MLKL mediates intracellular and intracellular ion imbalance leading to lytic cell death. Some studies have shown that MLKL takes up a large amount of Na^+^ during the process of mediating necroptosis [36,52], and in many cases cell lines that undergo necroptosis will take up a large amount of Na^+^ instead of Ca^2+^ [52]. These was consistent with the functionality of our unclipped form of AjMLKL. But some studies have shown that a large amount of Ca^2+^ influx occurs during necroptosis, and the Ca^2+^ channel is a downstream signal of MLKL and participates in mediating necroptosis [37,53]. However, contradictory to this is that blocking Ca^2+^ or Na^+^ channels, necroptosis signals cannot be specifically inhibited, and this can only be achieved under specific experimental conditions or in specifically treated cell lines [37,53]. All these novel finding implied that AjMLKL processing by the ‘early inflammasome’ appeared to be an evolutionary precursor of the real GSDM-mediated pyroptosis in higher organisms, and that different necrotic cell deaths might be combined together and segregated further at early stages of species evolution.

## Materials and Methods

### Animals

Healthy adult sea cucumbers (*A. japonicus*) were weighed 105 ± 12 g were collected from the Dalian Pacific Aquaculture Company (Dalian, China) and temporarily maintained in 30 L of aerated natural seawater (salinity of 28, temperature of 16 ± 1 °C) continuously for 5 days. The sea cucumbers in the breeding system were fed evenly every 2 days with feed containing 50% starch, 20% sea mud, 8% fish meal, and 22% seaweed powder. The feeding amount was about 10% of the total sea cucumber in the system. After feeding, the residue and residual bait were removed.

### Bacterial immersion infection

The *V. splendidus*-related strain AJ01 used in this study was isolated from diseased *A. japonicus*. The bacterium was identified by 16S rDNA sequence analysis, and its pathogenicity was determined in our previous study [78,79]. AJ01 was cultured at 28 °C in 2216E medium consisting of 5 g/L tryptone, 1 g/L yeast extract and 0.01 g/L FePO4 in aged seawater. For immersion infection, AJ01 cells were collected by centrifugation at 5000 ×g for 10 min and resuspended in filtered seawater. Sea cucumbers were randomly divided into four tanks each containing 10 individuals. The three experimental groups were infected by immersion with live AJ01 at a final concentration of 1 × 10^7^, 1 × 10^8^, or 1 × 10^9^ CFU/mL. The untreated group served as the control. After immersion infection for 0, 3, 6, and 12 h, coelomic fluids were collected from each individual in each group, passed through a 200 mm sterile nylon mesh, and centrifuged at 800 ×g for 5 min. Three biological replicates were conducted for each sampling point and used for subsequent related experimental analysis.

### Cell culture

The primary coelomocytes were prepared according to our previous work [80]. In brief, the coelomic fluids were filtered through a 200 Mesh Cell Cribble to remove large tissue debris. The cells were washed twice with isotonic buffer (0.001 M EGTA, 0.53 M NaCl, and 0.01 M Tris-HCl; pH 7.6) and resuspended in Leibovitz’s L-15 cell culture medium (Invitrogen, USA) supplemented with penicillin (100 U/mL) and streptomycin sulfate (100 mg/mL) with a final concentration of 1 × 10^6^ cells/mL. NaCl (0.39 M) was added to adjust the osmotic pressure to 780 mmol/L. The cells were dispensed in a 24-well culture microplate at 500 mL per well and preincubated in 16 °C overnight for subsequent related experimental analysis.

### Cell survival assay and membrane leakage assay

Cell survival assay and cell membrane leakage assay were performed using the CellTiter-Glo Luminescent Cell Viability Assay kit and CytoTox-Glo Cytotoxicity Assay kit, respectively, according to the manufacturer’s instructions (Promega). Luminescence was recorded with a Tecan GENios Pro Plate Reader.

### Coelomocyte lytic cell death assay by flow cytometry

Analysis of changes in necrotic coelomocytes after different treatments was performed by flow cytometry. Coelomocytes from different treatments were first harvested, double washed by using PBS and stained using the Annexin V-FITC/PI assay kit (Beyotime Biotechnology, China) based on the manufacturer’s protocols. A total of 10,000 stained coelomocytes were analyzed with a FACS Aria II flow cytometer (Becton Dickinson Biosciences, NJ, USA). Coelomocytes without any treatment were first collected as the blank group, gated and cleared of cell debris, and then the cells were single stained with FITC\PI, respectively, to adjust compensation. The coelomocytes were then double stained with Annexin V-Fluorescein Isothiocyanate (FITC)/PI and those stained at the same time were determined to be lytic cell death.

### RNA isolation and real-time quantitative PCR

Isolation of total RNA was performed using RNAiso Plus (TaKaRa) and synthesize cDNA was used PrimeScript RT kit and gDNA Eraser (TaKaRa). Applied Biosystem 7500 real-time quantitative pcr system (Thermo Fisher Scientific) was used to quantify mRNA expression. Specificity primers are presented on S1 Table. Ajβ-actin was identified used as a housekeeping gene for standardization of target quantification. The final volume of each reaction was 20 μL, which contained 6 μL of RNase-free H_2_O, 1 μL of each primer (10 μM), 2 μL of cDNA and 10 μL of SYBR Green PCR Master Mix (TaKaRa). Amplification was performed as described below: denaturation at 94°C for 2 min, followed by 40 cycles of 94°C for 15 s, 60°C for 20 s, and 72°C for 30 s. Melting curve analysis was performed after the cycling phase. For profiling of the Expression in each gene level, the 2^-ΔΔCT^ methodology was used [81].

### Gene cloning

The RNA was extracted from the coelomocytes of *A. japonicus* by using the RNAiso plus reagent (TaKaRa) and treated with the RNase-free DNase I (TaKaRa) to remove the genomic DNA. The first-strand cDNA was synthesized according to the Primescript II 1st cDNA Synthesis Kit (TaKaRa) following the manufacturer’s protocol. The gene specific primers of the AjMLKL gene (S1 Table) were designed based on the corresponding unigenes in the transcriptome data [82]. The full-length cDNA sequence of the AjMLKL was cloned subsequently by using the 3′,5′-Full RACE Kit (TaKaRa) following the manufacturer’s instructions. The desired PCR products were purified and cloned into the pMD19-T simple vector (TaKaRa). The ligation product was transformed into *Escherichia coli*-DH5α (TaKaRa), and the three positive clones for each product were sequenced at Sangon (Shanghai, China). The gene of AjNLRC4 and AjCASP-1 were generated according to our previous work [60,61]. Truncation mutants were constructed by polymerase chain reaction (PCR) cloning. Point mutations were constructed by using the QuickChange Site-Directed Mutagenesis Kit (Stratagene).

### Bioinformatics analysis

Phylogenetic trees were constructed by using MEGA 5.0 with the neighbor-joining or maximum likelihood (ML) method [83]. The potential functional motifs were predicted using the Pfam 31.0 and Conserved Domains Database of NCBI. The domain structures of AjNLRC4, AjCASP-1, AjMLKL were analyzed using AlphaFold2. The tertiary structural figures were reviewed and colored in PyMOL software.

### Purification of recombinant proteins

The specific primers for AjMLKL-4HB-F, AjMLKL-4HB-R, Cleaved AjMLKL-4HB-F, Cleaved AjMLKL-4HB-R, Cleaved AjMLKL-F and Cleaved AjMLKL-R (S1 Table) were used to amplify the different fragment of AjMLKL and Cleaved AjMLKL. Recombinant different fragment of AjMLKL and Cleaved AjMLKL were generated according to our previous work [60]. The concentration of the recombinant protein was quantified using the bicinchoninic acid method (Sangon Biotechnology, Shanghai). For futher Protein‐lipid overlay assay and liposome leakage assay. Similarly, the sequences of AjNLRC4-N were also amplified with the primers in S1 Table and cloned into the PGEX4T-1 vector to generate recombinant proteins. Purified GST-tagged AjNLRC4-N and His-tagged different fragment of AjMLKL were used for further functional analysis. To prepare active forms of recombinant AjCASP-1, The purified AjCASP-1 were diluted to 100 μg/ml in a buffer composed of 150 mM NaCl, 100 mM Hepes, 0.1% CHAPS, 10 mM dithiothreitol (DTT), 1% Triton X-100, and 10% sucrose (pH 7.5) and then mixed overnight at room temperature, as previously described [84].

### Antibody preparation

The AjMLKL, AjRIPK3 polyclonal antibody was prepared as described in previously described [60]. A 4-week-old mouse was intradermally injected with a mixture, which contained 100 μg purified His-tagged recombinant protein and an equal volume of complete Freund’s adjuvant (Promega). After two weeks, the mouse was intramuscularly boosted twice with 100μg of His-tagged recombinant protein mixed with an equal volume of in-complete Freund’s adjuvant at 1-week interval. The antiserum was stored at −80 °C for subsequent experiments. The AjNLRC4 and AjCASP-1 polyclonal antibody were produced as in Chen et al. (2021) [61] and Shao et al. (2019) [60].

For the preparation of AjCASP-1 polyclonal antibody, a rabbit weighing 3 kg was immunized with recombinant AjCASP-1 protein according to the following procedure [85]. Afterward, 2 mL of the AjCASP-1 protein dissolved in phosphate buffered saline (PBS) (0.3 mg mL^−1^) was emulsified with an equal volume of Freund’s complete adjuvant (Promega) and was intracutaneously injected into the rabbit. The rabbit was immunized intensively by injecting 1.5 mL of the AjCASP-1 protein concentration mixed with equal volume of incomplete Freund’s adjuvant on 7 and 14 days after the first immunization. Finally, the rabbit was bled from an ear vein on 22 days after being treated for 1-day fasting, and the antisera were collected through centrifugation of the blood samples. The antiserum was stored at −80 °C for subsequent experiments. The other antibodies used were as follows: mouse anti–β-actin monoclonal antibody (purchased from Abcam). HRP-conjugated anti-mouse/rabbit IgG (D110087; D110058) secondary antibodies were sourced by Sangon (China).

### Phosphorylation analysis of AjMLKL

Freshly coelomocytes were collected from *A. japonicus* stimulated by AJ01(1 × 10^8^ CFU/mL) for 0 h,3 h and 6 h were used to extract protein for protein quantification and phosphorylation analysis. Phosphorylation of AjMLKL during AJ01 infection was analyzed using phos-tag method SDS-PAGE (Wako, Japan) following vendor protocol. The coelomocytes proteins were separated on 10% SDS-PAGE with phos-tag molecules. Thereafter, the gel was washed twice with transfer buffer containing 10 mM EDTA (39 mM glycine, 48 mM Tris), and then washed once in transfer buffer without EDTA. The gel was then transferred onto a polyvinylidene fluoride (PVDF) membrane. The membrane was blocked with 5% skim milk in TBST (20 mM Tris-HCl, 150 mM NaCl, and 0.05% Tween-20) at 37 °C for 1 h before being incubated with specific polyclonal antibodies (usually dilution to 1:500 in 5% skim milk) at room temperature for 2 h. Subsequently, the membranes were washed three times with TBST before being incubated with HRP-linked secondary antibodies (Sangon, Shanghai, China) for 1.5 h at RT. The signals on the membranes were detected by reacting with Super-signal West Pico Chemiluminescent Substrate (Sangon, Shanghai, China) and exposed to the gel imaging recorder (Omega, USA).

### RNA interference

Specific siRNAs for AjNLRC4, AjCASP-1, and AjMLKL were made synthetically by GenePharma (S1 Table). The control siRNA (NC), which did not specifically target any of the genes in the sea cucumber transcriptome, was used as a control (S1 Table). All siRNAs were dissolved the siRNAs in RNase-free H_2_O and prepared a 20 μM stock solution. Two gene-specific siRNAs were mixed for RNA interference assay, and 100 μL of the transfection solution consisting of 5 μL of each siRNA, 10 μL of Lipo6000 transfection reagent (Invitrogen, USA), and 80 μL of phosphate buffer (PBS) was injected through the tentacles of the sea cucumbers in the interference group. The control group was injected with NC siRNA under the same conditions. After transfection for 24 h, the control and treated coelomocytes were harvested for qPCR, Membrane Leakage Assay, ATP cell viability, Western blot, immunoprecipitation and immunofluorescence assays.

### Overexpression assay

AjNLRC4, AjCASP-1 were overexpressed according to our previous work [61]. In brief, the ORFs without encoding signal peptide of each gene were amplified by primers in (S1 Table). The product fragment of the PCR was subsequently ligated to the pET-32a+ vector and used for mRNA synthesis in vitro according to a previously described method46. A total of 300 μg of mRNA was injected into each group and incubated up to 24 h. Then the efficiency of overexpression was subsequently tested by Western blot and qPCR.

### Western blotting

For detailed western blot methods, please refer to our previous research [61]. The primary antibodies used in this experiment and their dilutions are shown below: AjNLRC4 antibody (diluted 1:400), AjCASP-1 antibody (diluted 1:500), β-actin antibody (diluted 1:3000), AjMLKL antibody (diluted 1:400) and AjRIPK3 antibody (diluted 1:400). The HRP-conjugated anti-mouse/rabbit IgG was used as secondary antibodies (diluted 1:3000). The blot band intensity was estimated with the ImageJ software (NIH, Bethesda, MD, USA).

### Intracellular AJ01 survival assay

Intracellular AJ01 survival measurements were made according to a former description [61]. coelomocytes were plated at a density of 5 × 10^4^ cells per well in 24-well plates. Each well was infected with AJ01 at a final concentration of 10^8^ CFU/ml for 6 h. Coelomocytes were then washed with PBS and replaced with coelomocytes culture medium containing 50 μg/ml gentamicin, and co-incubation was continued for 1.5 h. After washing again with PBS for 3 times, the cells were lysed by adding Triton X-100 to release intracellular AJ01, then the released AJ01 was diluted and plated on 2216E plates, incubated overnight at 28 °C and counted by CFU assay.

### BLI assay

Binding kinetics of AjMLKL\AjVEGF and GW807642X were determined by BLI (ForteBio Octet RED96e) as previously described with minor modification [86]. Briefly, the His-tag AjMLKL\AjVEGF protein were immobilized to His biosensors after prewetting with 1 × kinetic buffer (ForteBio). The equilibrated His biosensors were incubated with GW807642X diluted at indicated concentrations. The association and dissociation process were then per- formed for 300 or 400 s at 25°C with shaking, respectively. All these data were recorded and analyzed using Octet RED96e data analysis software.

### Pull-down and coimmunoprecipitation (Co-IP) assay

The interaction between AjNLRC4 and AjCASP-1 was demonstrated by pull down assay. Purified GST-tagged AjNLRC4-Ig-like and Ig (AjNLRC4-N) (200 μg) or GST-tagged AjNLRC4-IN (200 μg) or GST-tagged AjMLKL with His-tagged AjCASP-1 or rHis-AjCASP-1-CASc or rHis-AjCASP-1-N, respectively, to be incubated overnight at 4°C. Then, 100 μL of GST-bound resin was added to each mix and incubated for 50 min at 4 °C. The collected resin was washed five times by PBS followed by elution of the combined proteins with Elution Buffer 1 (10 mM reduced glutathione, 50 mM Tris-HCl, pH 8.0). The reverse pull-down assay was performed with His-tag protein as bait protein. The binding proteins were eluted by Elution buffer 2 (20 mM Tris-HCl, 0.5 M NaCl, 1 M imidazole, pH 8.0). Detection of bound proteins using SDS-PAGE. The interactions of AjNLRC4 with AjCASP-1, AjMLKL, and AjCASP-1 were determined by Co-IP assay as previously described [87]. AJ01-infected or uninfected coelomocytes were lysed in lysis buffer (150 mM NaCl, 1.0% Nonident-P40, 0.1% SDS, 50 mM Tris, pH 8.0), the supernatant was centrifuged to extract proteins, and protein A+G (Beyotime, China) was added for 10 min to remove non-specific binding proteins. Subsequently, antibodies specific for AjNLRC4 or AjCASP-1 (AjMLKL-like or AjCASP-1), respectively, were added and incubated at room temperature for 3 h. Protein A+G was added again and incubated at 4°C for 3 h. The pellet was washed 5 times with TBS. The obtained pellets (bound protein, Ab and protein A+G) were subjected to Western blotting analysis.

### Mass spectrometry

Infected coelomocytes were lysed with lysis buffer (50mM pH8.0 Tris-HCl; 150 mM NaCl; 0.5% NP40; 1 mM EDTA; protease inhibitors (Beyotime, P1045, CN)) on ice for 10 min, then incubated with specific AjMLKL polyclonal antibody coated protein G dynabeads (10003D, Thermo Scientific, US) overnight for immunoprecipitation. Proteins pulled down by protein G dynabeads were washed by SDT buffer (4% SDS; 100 mM pH8.0 Tris-HCl; 1 mM DTT). Significantly different bands were detected using silver staining SDS-PAGE and further characterized by Mass spectrum analyses by Q Exactive (Thermo Fisher, US).

### CASP activity analysis *in vivo*

CASP activity was performed with a human CASP-1 chromogenic substrate (Ac-YVAD-pNA) using a CASPASE activity assay kit (Beyotime Biotechnology, China) according to the manufacturer’s protocol. Lysis of differently treated cells with caspase cell lysate. The lysis products were added to caspase assay buffer which contains 100 mM Ac-YVAD-pNA. After incubation at 37 °C for 2 h, the cleavage of caspase-type-specific substrate emitted a fluorescent signal measured at 405 nm with a microplate reader (Thermo Scientific). Experiments were repeated in triplicate and Ac-YVAD-pNA as a reference.

### AjCASP-1 oligomer cross-linking

For AjCASP-1 oligomer cross-linking, coelomocytes were cultured in six-well plates and infected with 10^8^ CFU/ml AJ01 for 3 h. The supernatant was removed, lysed with 0.5% Triton X-100 in PBS buffer, and collect the supernatant through centrifugation at 6797 x g for 15 min at 4°C and transferred to a new tube (soluble fraction). The pellet insoluble in Triton X-100 were washed several times and esuspended in 200 μL of PBS and 4 mM sodium dextran sulphate was added and the pellet was cross-linked for 30 minutes at room temperature. The cross-linked pellets were centrifuged at 6797 x g for 15 minutes to dissolve in the SDS sample buffer directly.

### Field-emission gun scanning electron microscopy (FEG-SEM) assay

Coelomocytes from the different treatments would be centrifuged for 10 minutes at 4500 rpm, resuspended in 2.5% glutaraldehyde fixative (Shanghai Yuanye) and immobilized at 4°C for 3 hours. Following centrifugation, coelomocytes were washed and resuspended in PBS. Droplets of suspension (10 μL) were added to cell-adherent slides and left overnight at 4°C. Cells were then dehydrated in a series of 30%, 50%, 70%, 80% and 90% ethanol over 15 minutes each in successive series. A graded concentration of a mixture of ethanol and tert-butanol was then used to replace the ethanol in the samples. Samples were dried for 24 hours in a freeze dryer (Alpha 1-4LD plus, Germany), and gold nanoparticles were sprayed under an ion sputterer and imaged with a FEG-SEM (HITACHI S-3400, Japan).

### *In vitro* MLKL cleavage by recombinant CASP-1

*In vitro* MLKL cleavage assay was performed to determine AjCASP-1 cleavage specificity toward AjMLKL. Briefly, five micrograms of wild type or mutant AjMLKL were incubated with different micrograms of purified activated form of AjCASP-1(p20/p10) (0, 5, 10,15,20μg) or unactivated form of rAjCASP-1^1-299aa^ in a final volume of 25μL reaction containing 50 mM HEPES (pH 7.5), 3 mM EDTA, 150 mM NaCl, 0.005% (vol/vol) Tween-20, and 10 mM DTT for 3 h or with 10 micrograms of activated forms of AjCASP-1 for different hours (0, 1, 2, 4 and 6 h). Where CASP inhibitors were involved, 10 micrograms of activated forms of AjCASP-1 were pre-incubated with the inhibitors for 1 h, and then incubated with AjMLKL for 3 h. To examine rAjMLKL-4HB domain was cleavage by HsCASP-1, rAjMLKL-4HB was incubated with 5 U of rHsCASP1 (Enzo Life Sciences, Villeurbanne, France) at 37 °C for 1 h in a 25 μl reaction system. The cleavage products were isolated by SDS-PAGE and detected by Coomassie blue staining and immunoblotting

### Edman sequencing

Edman sequencing was performed as previously reported [88]. Briefly, rAjMLKL was incubated with activated rAjCASP-1 at 37 °C for 3 h, followed by 12% SDS-PAGE to separate the cleaved fragments. The cleaved bands were cut out and subjected to Edman degradation in a PPSQ-33A automated protein sequencer (Shimadzu, Kyoto, Japan).

### Protein-lipid overlay assay

The recombinant proteins of MLKL, cleaved AjMLKL, AjMLKL-4HB domain and 4HB^18-134^ domain were diluted to 200 ng/ml. we analyzed protein–lipid interactions with commercial lipid strips (Echelon Biosciences) according to the manufacturer’s instructions. The different fragment of recombinant proteins of AjMLKL were diluted with washing buffer (PBST with 3% fatty acid free BSA) to 200 ng/mL. Then, using 1× PBS containing 0.1% Tween-20 (PBST) and 3% fatty acid-free bovine serum albumin (BSA; Biotopped) to co-incubated with the lipid strips for 1 h at room temperature. The strips were then incubated with the above treated proteins for 1 h at room temperature, followed by three washes with PBST buffer. The lipid strips were then co-incubated with 1× PBS containing 0.1% Tween-20 (PBST) and 3% fatty acid-free bovine serum albumin (BSA; Biopped) for 1 h at room temperature. The AjMLKL antibody was diluted 1:2000 in PBST buffer containing 3% BSA and incubated with the lipid strips for 1 hour at room temperature. After three subsequent washes with PBST buffer, the secondary antibody labeled with HRP was again incubated with the lipid strip for 1 h. The proteins were made visual with High-sig ECL Western blotting substrate after three washes with PBST buffer and exposed on X-ray film.

### Fractionation membrane proteins by phase separation

Fractionation by Phase Separation as previously described [38].Triton X-114 lysis buffer was used to perform the phase separation assay and overviewed in Fig 2O. The pellets from treated cells were resuspended in 5X volume of Triton X‐114 lysis buffer (20 mM HEPES, pH 7.4, 150 mM NaCl, 2% Triton X‐114, and complete protease inhibitor [Roche]) and incubated on ice for 30 min. The cell lysate was centrifuged at 15,000 × g at 4°C for 10 min, and then the supernatant was harvested as the detergent soluble fraction. After warming at 30°C for 3 min, the detergent soluble fraction was centrifuged at 1,500 × g for 5 min at room temperature. The aqueous layer was collected then re‐centrifuged at 1,500 × g for 5 min to remove the contamination from the detergent enriched layer and saved as the aqueous faction (Aq). The detergent enriched layer was diluted with basal buffer (20 mM HEPES, pH 7.4, 150 mM NaCl) to the same volume of the detergent soluble fraction and re‐centrifuged at 1,500 × g for 5 min. The washed detergent enriched layer was diluted with the basal buffer to the same volume as the aqueous faction and saved as the detergent fraction (Det).

### Preparation of liposomes

Natural and synthetic lipid products (Avanti Polar Lipids) were dissolved as a 20mM solution in chloroform. The lipid films (2 μmol) of POPC and POPE (with molar ratio 4:1) with or without other phospho‐lipids were obtained by evaporating the solvent using dry nitrogen. The lipid films were hydrated with 300 μl buffer L (20 mM HEPES, pH 7.4, 100 mM NaCl) and liposomes were prepared by extrusion 24 times through a 100‐nm polycarbonate membrane followed by dilution in 1 ml buffer L. To prepare Tb3+ encapsulated liposomes, the lipid films were hydrated with 300 μl buffer TL (20 mM HEPES, pH 7.4, 100 mM NaCl, 50 mM sodium citrate, 15 mM TbCl3). After extrusion, the liposomes were washed twice to remove external Tb3+ with 1 ml buffer TW (20 mM HEPES, pH 7.4, 50 mM NaCl, 50 mM sodium citrate) by centrifugation in a TLA‐55 rotor (Beckman) at 4°C for 20 min at 100,000 × g and then re‐suspended in 1 ml buffer L. To prepare fluorescein labeled dextran (Invitrogen) encapsulated liposomes, the lipid films were hydrated with 300 μl of 1 mg/ml dextran in buffer L. After extrusion, the liposomes were washed twice to remove external dextran with 1 ml buffer L by centrifugation and then re‐suspended in 1 ml buffer L. All the liposomes were stored at 4°C and used within 24 h.

### Liposome sedimentation assay

AjMLKL, cleaved AjMLKL^18-491^, 4HB domain of AjMLKL and 4HB^18-134aa^recombinant proteins (10 μM concentration) were incubated with liposomes (1 mM lipid concentration) at room temperature for 30min in 0.1ml buffer L. The liposomes were pelleted by centrifugation in a TLA‐55 rotor (Beckman) at 4°C for 20 min at 100,000 × g. The supernatant (S) containing the free protein that is not bound to liposomes was collected. The liposome pellets (P) were washed twice with 1 ml buffer L by centrifugation and then re‐suspended in 0.1 ml buffer L. The MLKL protein in both S and P fractions were analyzed by SDS‐PAGE followed by coomassie blue staining.

### Liposome sedimentation assay and leakage assay

Liposome Sedimentation Assay and Leakage Assay based on the previous report [38] Natural and synthetic lipid products used for liposome preparation were from Avanti The liposomes were prepared using Avanti Mini-Extruder according to the manufacturer’s instructions. Liposomes prepared within 24 h were aliquoted and used for liposome sedimentation assay and leakage assay. For liposome sedimentation assay, 10 mM AjMLKL, cleaved AjMLKL, AjMLKL-4HB domain or 4HB^18-134^ domain recombinant proteins were incubated with 1 mM (lipid concentration) liposomes for 30 min, respectively, and pelleted by centrifugation at 4 °C for 20 min at 100,000 g. The co-sedimented proteins were analyzed by SDS-PAGE followed by Coomassie blue staining. For liposome leakage assay, the released fluorescence was recorded with a Tecan GENios Pro Plate Reader.

### Liposome leakage assay

For the time course liposome leakage assay, aliquots of Tb^3+^ encapsulated liposomes with 300μM total lipid concentration were diluted in 100 μl buffer L supplemented with 50 μM of DPA. When 0.5 μM of recombinant proteins were added, the emission spectrum was measured as F_t0_. The fluorescence was continuously recorded as Ft for 10 min at 15 s intervals, then 0.1% Triton X-100 was added to achieve complete release of Tb^3+^. The mean values of the top‐three fluorescence reads after adding Triton X-100 were calculated as F_t100_. At each time point, the percentage of liposome leakage is defined as:

Leakage (t) (%) = [(F_t_‐F_t0_)/ (F_t100_‐F_t0_)] × 100

### Bacterial growth assay

Colony-forming unit assays and turbidimetry were used to measure bacterial growth as previously described [89]. Briefly, for turbidimetry, bacteria were diluted (1:100) in bacterial culture medium following treatment and incubated with discontinuous shaking at 37 °C in a 200 μl volume in flat-bottomed 96-well plates. Growth curves were monitored by reading absorbance at 600 nm over 16 h using a Spectra MAX 340 (Molecular Devices) or Synergy H4 Hybrid Multi-Mode Microplate Reader (BioTek). The time until the growth curves reached a threshold OD_600_ of 0.05 above background was defined as the T_*threshold*_. The ratio of T_*threshold*_ (treated): T_*threshold*_ (untreated) was used to quantify the change in bacterial growth.

### LIVE/DEAD assay

Bacterial viability was assessed using the bacterial LIVE/DEAD assay (Invitrogen), following the manufacturer’s recommendations. Briefly, bacteria were treated in the presence of 5 μM Syto-9 (Invitrogen) and 15 μM propidium iodide (Invitrogen). Treatment with 70% isopropanol served as a positive control. Fluorescence was visualized by confocal microscopy.

### Immunofluorescence analysis

To analyze the effect of AjNLRC4/AjCASP-1 on the migration of AjMLKL from cytoplasm to membrane during AJ01 infection, the distribution of AjMLKL was analyzed by immunofluorescence. For detailed labeling staining methods, please refer to our previous research [61]. For time-lapse microscopy, cells were plated onto a 35-mm glass bottom dish (Nest). To monitor the intracellular ion concentration, coelomocytes were loaded with sodium indicator ANG-2, potassium indicator APG-2 and calcium indicator Fluo-4 respectively according to the manufacturer’s protocol. PI (5 ng/ml) was added to medium for monitoring cell membrane integrity. Imaging was carried out using Zeiss LSM 780. Unprocessed images were analyzed by ImageJ software.

### Statistical analysis

Data were presented as the mean ± the SD of at least three replicates. One-way ANOVA was used for multiple comparisons, with different lowercase letters indicating significant differences (*P* < 0.05) in the ANOVA analysis. Significance differences for paired comparisons were analyzed using Student’s t test. The statistical analysis used for each experiment was described in the figure legends. Densitometry analyses of western blotting bands were based on three independent replicates using Quantity One software. All statistical analyses were performed using GraphPad Prism 9.5.1 software. Tables listing primers are provided in S1 Table and S1 Data.

### Data, materials, and software availability

Data were presented as the mean ± the SD of at least three replicates. One-way ANOVA was used for multiple comparisons, with different lowercase letters indicating significant differences (*P* < 0.05) in the ANOVA analysis. Significance differences for paired comparisons were analyzed using Student’s t test.

### Limitations of the study

Owing to the lack of efficient genetic editing technique in lower sea cucumber, we could not use knockout cells to validate our results. Therefore, we perform the knock-down and inhibitor treatment with the most perfect control as alternative. Another limitation of this study is that because of the absence of gasdermin family members in sea cucumber, cell death in this study could not be defined as pyroptosis or necroptosis. Therefore, we used the lytic coelomocyte death as the mixed of the two similar deaths.

## Supporting information

S1 Fig Sequence characters of AjMLKL.
(A) Amino acid sequences of the AjMLKL. (B) The domain architecture of AjMLKL was predicted by SMART. (C) Phylogenetic analysis of MLKL from A. japonicus and other species. The neighbor-joining tree was constructed using MEGA 5.2 program, with bootstraps of 1000 to test the reproducibility. MLKL from M. japonicus were labelled with a blue box. The GenBank accession number of each sequence was shown in the figure. (D) The specific Ab detection of AjMLKL in coelomocytes. AjMLKL protein expression in the sea cucumber coelomocytes under different treatment, as detected by western blotting with anti-AjMLKL serum as the primary antibody to clarify the specificity of the AjMLKL antibody. (E) Gray value analysis of AjMLKL protein expression levels at different time points after AJ01 infection.
(TIF)

S2 Fig AjRIPK3 not interacting with AjMLKL.
(A) The domain architecture of AjRIPK3-like, HsRIPK3 and MmRIPK3 were predicted by SMART. (B) The specific Ab detection of AjRIPK3 in coelomocytes. AjRIPK3 protein expression in the sea cucumber coelomocytes, as detected by western blotting with anti-AjRIPK3 serum as the primary antibody to clarify the specificity of the AjRIPK3 antibody. (C and D) Co-IP assays to analyze the interaction between AjMLKL with AjRIPK3 in vivo. (E-F) The efficiency of AjMLKL-RNAi in coelomocytes was determined using qPCR (E) and western blotting analysis (F). The graphs are representative of three independent assays, and the proportions were calculated from those three assays, *p < 0.05.
(TIF)

S3 Fig GW806742X specifically targets AjMLKL and inhibits the migration of AjMLKL and its cleavage products to the cell membrane.
(A-B) Affinity of AjMLKL/AjVEGF to GW806742X measured by BLI. The affinity curves of AjMLKL (A) or AjVEGF (B) binding to GW806742X are expressed in nanometers (response unit) versus time. The gradient concentrations (25, 50, 100, and 200 nM) of GW806742X (C211A) were incubated with AjMLKL/AjVEGF. (C) Sea cucumbers were challenged with 108CFU/mL AJ01. And then, the membrane and cytoplasm proteins were extracted from the co elomocytes. AjMLKL and its cleavage products expression in the membrane and cytoplasm of coelomocytes was analyzed using western blotting at 0, 3, 6, and 12 h post-infection with 108CFU/mL AJ01. The panels show the statistical analysis of three independent experiments. *p < 0.05. (D) Sea cucumbers were treated with GW806742X for 3 h, and then challenged with 108CFU/mL AJ01 for 3 h, the membrane and cytoplasm proteins were extracted from the coelomocytes. AjMLKL and its cleavage products expression in the membrane and cytoplasm of coelomocytes was analyzed using western blotting. The panels show the statistical analysis of three independent experiments. *p < 0.05.
(TIF)

S4 Fig Determination of optimal inhibitor concentration.
(A) The red box representing potential CASP-1 cleavage motifs, the cleave sites is predicted by Peptide Cutter. (B) Coelomocytes from AJ01-infected different times sea cucumber were examined for AjCASP-1 activities. (C) rAjCASP-1 incubated with the chromogenic substrate Ac-YVAD-pNA and treatment with 25 μM Z-YVAD-FMK or DMSO (control) for 3 h to determine CASP-1 activity. (D) The effect of Z-YVAD-FMK on the cell viability of sea cucumbers. Sea cucumber coelomocytes were treated with increasing concentrations of Z-YVAD-FMK for 3 h.
(TIF)

S5 Fig HsCASP-1 cleaves AjMLKL-4HB.
His-tagged AjMLKL-4HB was incubated with human caspases (HsCASP1) for 30 min and then stained with coomassie blue.
(TIF)

S6 Fig Cleaved-AjMLKL through its 4HB domain engages bactericidal activities.
Escherichia coli expressing cleaved-AjMLKL-4HB (18-134 residues), cleaved-AjMLKL (18-491 residues) were grown on LB agar plates and induced with IPTG for 12 h at 16°C.
(TIF)

S7 Fig Effects of cleaved and uncleaved AjMLKL on intracellular ion homeostasis.
Intracellular ion concentration was monitored in uncleaved MLKL or cleaved MLKL overexpressed coelomocytes treated with AJ01. Sodium indicator (ANG-2), Calcium indicator (Fluo-4) and Potassium indicator (APG-2) were used. Trx-his Tag mRNA overexpressing coelomocytes were treated with AJ01 as a control. The coelocoelomocytes treated as above were collected and detected by flow cytometry.
(TIF)

S8 Fig Structural characterisation of the AjNLRC4 and AjCASP-1 proteins and interaction sites.
(A-D) Prediction of crystal structures of each domain of CASP-1 and tertiary structure of AjCASP-1 and HsCASP-1 by AlphaFold2 (E-H) Domain architecture and tertiary structure of AjNLRC4 and HsNLRC4 modeled by AlphaFold2. (I) Analysis of the structure of the Ig-like and Ig domain in AjNLRC4. The top five threading templates used by I-TASSER are 3alpA, 4fomA, 5zo1A, 6efzA, 1dgiR. (J and K) Amino acid sequences of the Ig-like and Ig domain of AjNLRC4, with the red box representing potential CASP-1 binding motifs.
(TIF)

S9 Fig Measurement of interference efficiency of AjNLRC4 or AjCASP-1 by specific siRNA transfection.
Sea cucumbers were transfected with specific AjNLRC4/AjCASP-1 siRNA for 24 h, respectively. The non-targeting siRNA (NC) was transfected as the control group. Then, 108 CFU/ml AJ01 at final concentration was added in the experimental and NC group for another 12 h. Finally, the relative expression of AjNLRC4 (a) or AjCASP-1 (b) in coelomocytes was determined at mRNA levels by using qPCR and at protein levels by western blotting analysis. It was found that after the above treatment, the mRNA and protein levels of AjNLRC4 were significantly decreased. Results are representative of at least three independent experiments, and error bars denote the SD of triplicate wells. *p < 0.05, **p < 0.01.
(TIF)

S10 Fig The efficiency of AjNLRC4/AjCASP-1 overexpression in coelomocytes, as determined using qPCR and western blotting analysis.
The Sea cucumbers were injected with AjNLRC4/AjCASP-1 mRNA for 24 h. The Trx-His tag mRNA was injected in as the control group. After the injection of AjNLRC4/AjCASP-1 mRNA, the mRNA and protein levels of AjNLRC4/AjCASP-1 mRNA were significantly up-regulated. Results are representative of at least three independent experiments, and error bars denote the SD of triplicate wells. *p < 0.05, **p < 0.01.
(TIF)

S1 Data The Numerical data used in all figures.
(XLSX)

S1 Table qPCR metadata for all qPCR assays.
(DOCX)
