# Supplementary figures and images for: Lytic coelomocyte death is tuned by cleavage but not phosphorylation of MLKL in echinoderms
Source: PLoS Pathog. 2025 Mar 14;21(3):e1012991. doi: 10.1371/journal.ppat.1012991 (PMC11932488; doi:10.1371/journal.ppat.1012991)

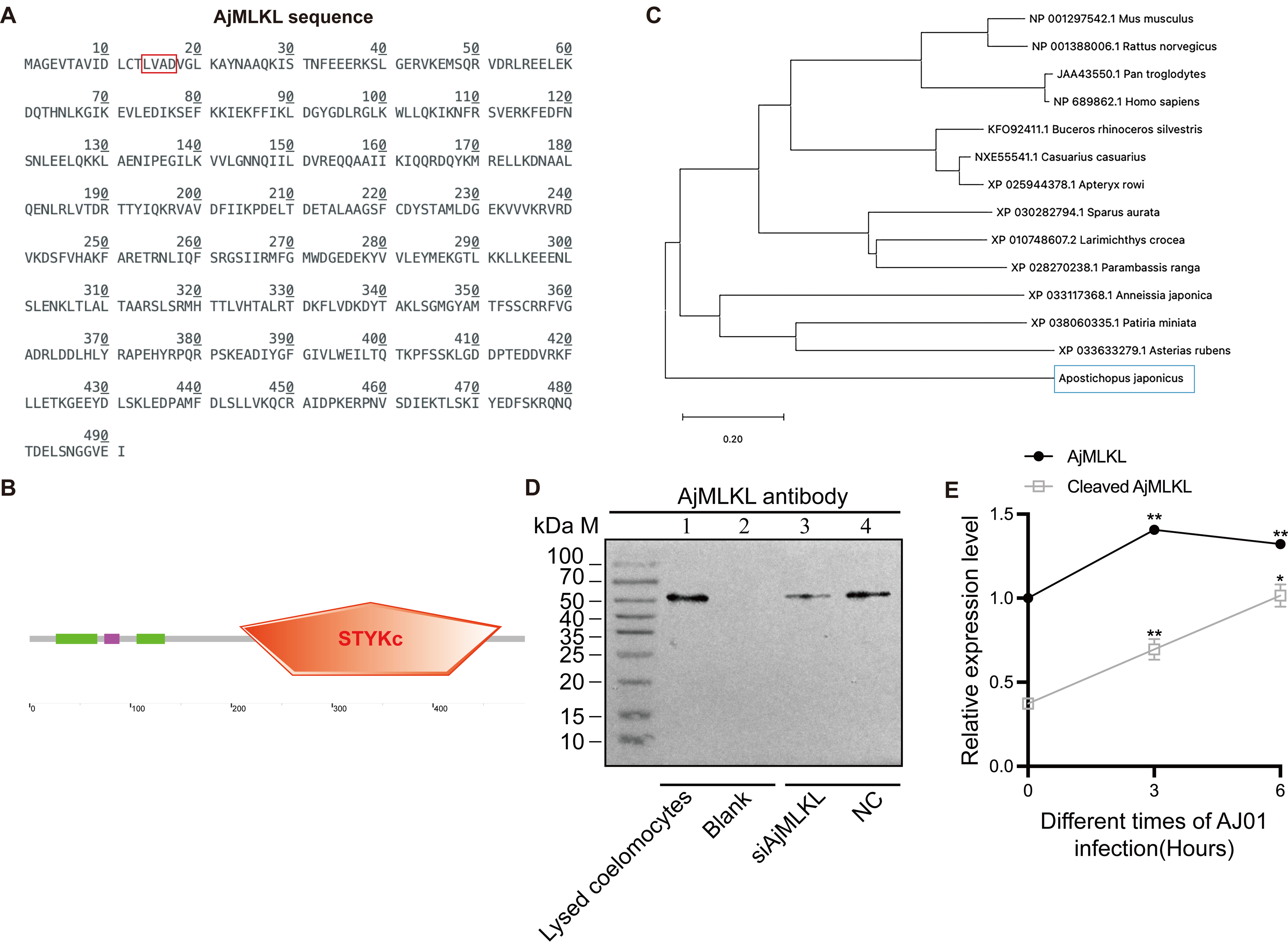

Supplement: S1 Fig — (A) Amino acid sequences of the AjMLKL. (B) The domain architecture of AjMLKL was predicted by SMART. (C) Phylogenetic analysis of MLKL from A. japonicus and other species. The neighbor-joining tree was constructed using MEGA 5.2 program, with bootstraps of 1000 to test the reproducibility. MLKL from M. japonicus were labelled with a blue box. The GenBank accession number of each sequence was shown in the figure. (D) The specific Ab detection of AjMLKL in coelomocytes. AjMLKL protein expression in the sea cucumber coelomocytes under different treatment, as detected by western blotting with anti-AjMLKL serum as the primary antibody to clarify the specificity of the AjMLKL antibody. (E) Gray value analysis of AjMLKL protein expression levels at different time points after AJ01 infection. (TIF) [file ppat.1012991.s001.tif]

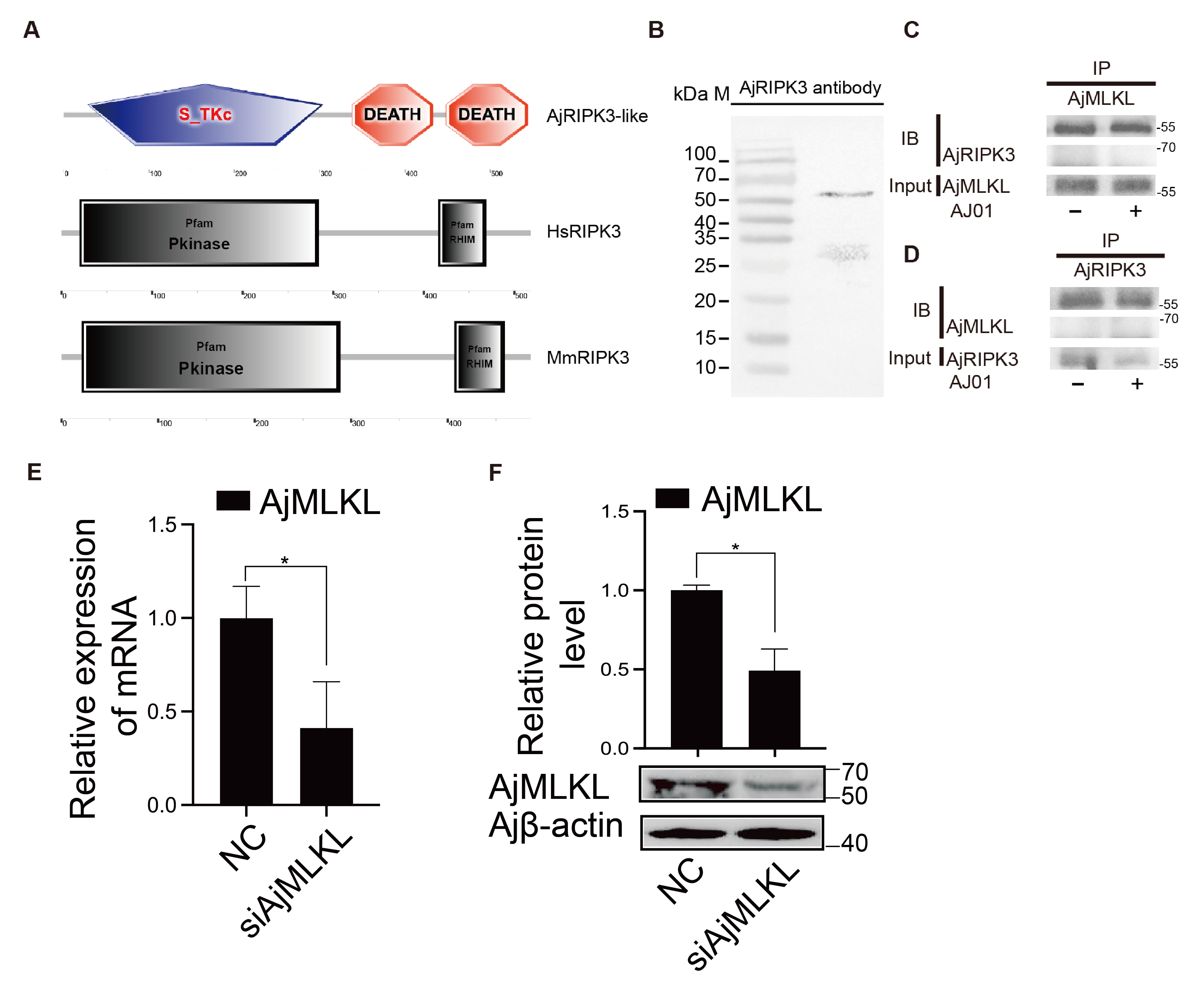

Supplement: S2 Fig — (A) The domain architecture of AjRIPK3-like, HsRIPK3 and MmRIPK3 were predicted by SMART. (B) The specific Ab detection of AjRIPK3 in coelomocytes. AjRIPK3 protein expression in the sea cucumber coelomocytes, as detected by western blotting with anti-AjRIPK3 serum as the primary antibody to clarify the specificity of the AjRIPK3 antibody. (C and D) Co-IP assays to analyze the interaction between AjMLKL with AjRIPK3 in vivo. (E-F) The efficiency of AjMLKL-RNAi in coelomocytes was determined using qPCR (E) and western blotting analysis (F). The graphs are representative of three independent assays, and the proportions were calculated from those three assays, *p < 0.05. (TIF) [file ppat.1012991.s002.tif]

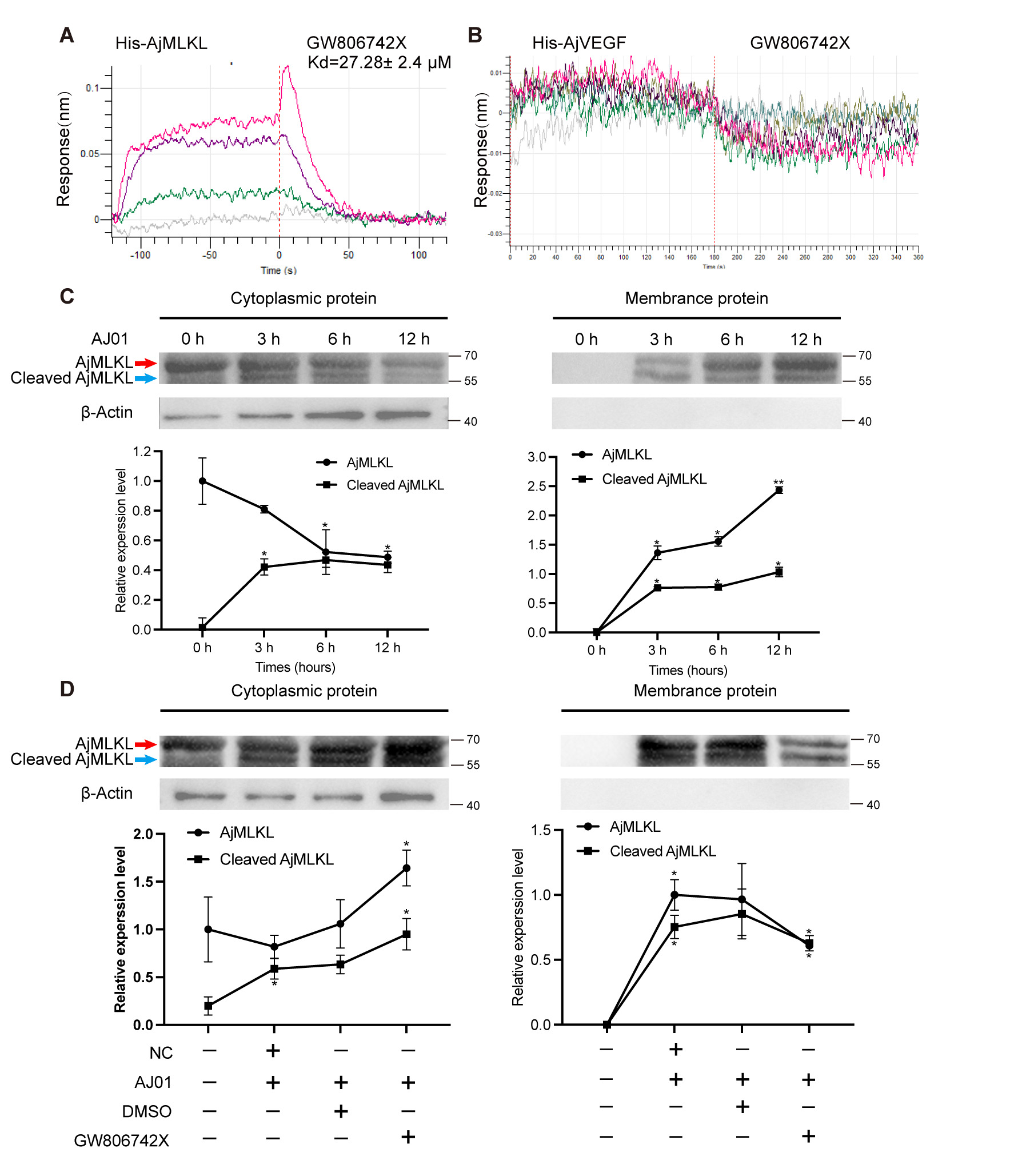

Supplement: S3 Fig — (A-B) Affinity of AjMLKL/AjVEGF to GW806742X measured by BLI. The affinity curves of AjMLKL (A) or AjVEGF (B) binding to GW806742X are expressed in nanometers (response unit) versus time. The gradient concentrations (25, 50, 100, and 200 nM) of GW806742X (C211A) were incubated with AjMLKL/AjVEGF. (C) Sea cucumbers were challenged with 108CFU/mL AJ01. And then, the membrane and cytoplasm proteins were extracted from the co elomocytes. AjMLKL and its cleavage products expression in the membrane and cytoplasm of coelomocytes was analyzed using western blotting at 0, 3, 6, and 12 h post-infection with 108CFU/mL AJ01. The panels show the statistical analysis of three independent experiments. *p < 0.05. (D) Sea cucumbers were treated with GW806742X for 3 h, and then challenged with 108CFU/mL AJ01 for 3 h, the membrane and cytoplasm proteins were extracted from the coelomocytes. AjMLKL and its cleavage products expression in the membrane and cytoplasm of coelomocytes was analyzed using western blotting. The panels show the statistical analysis of three independent experiments. *p < 0.05. (TIF) [file ppat.1012991.s003.tif]

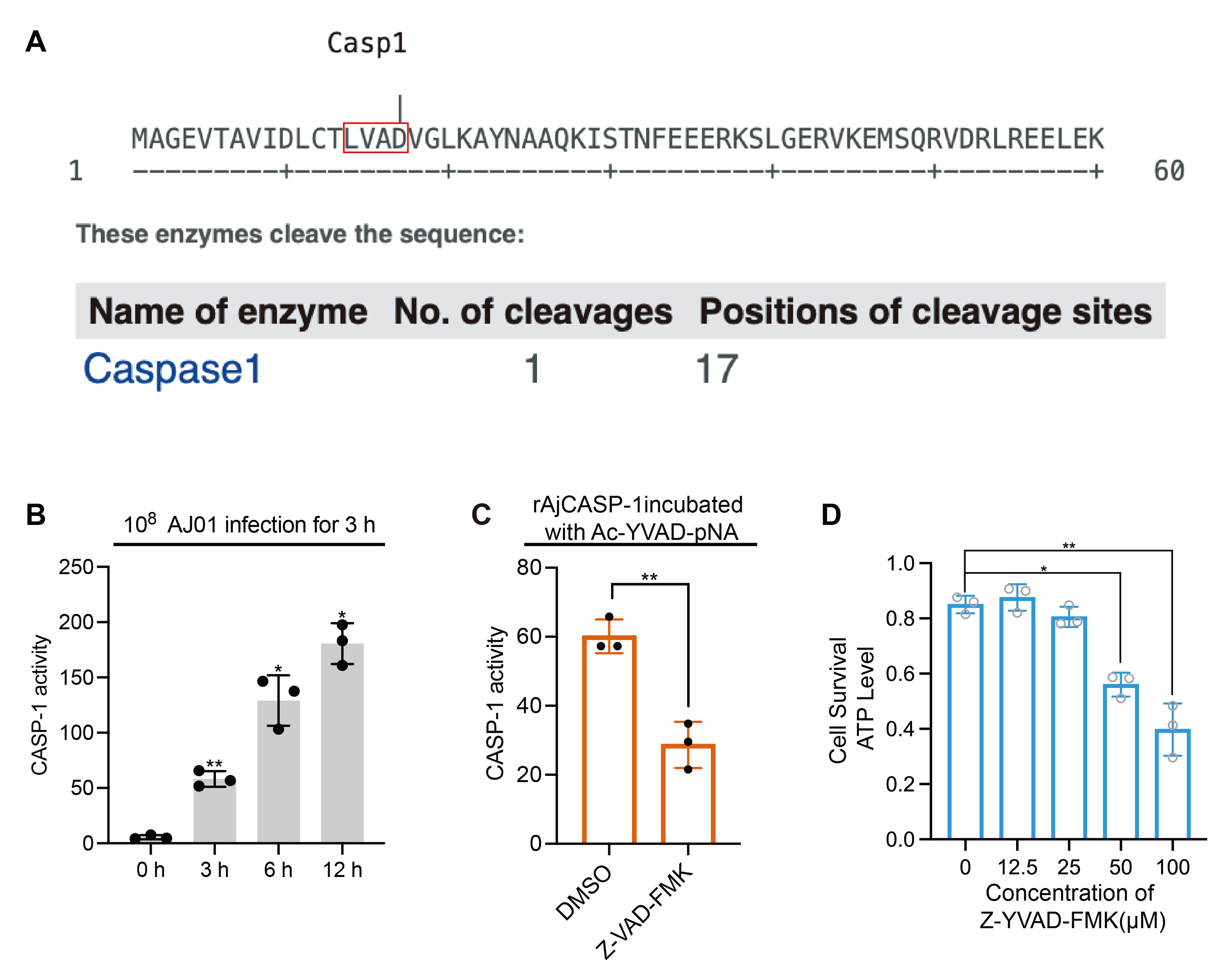

Supplement: S4 Fig — (A) The red box representing potential CASP-1 cleavage motifs, the cleave sites is predicted by Peptide Cutter. (B) Coelomocytes from AJ01-infected different times sea cucumber were examined for AjCASP-1 activities. (C) rAjCASP-1 incubated with the chromogenic substrate Ac-YVAD-pNA and treatment with 25 μM Z-YVAD-FMK or DMSO (control) for 3 h to determine CASP-1 activity. (D) The effect of Z-YVAD-FMK on the cell viability of sea cucumbers. Sea cucumber coelomocytes were treated with increasing concentrations of Z-YVAD-FMK for 3 h. (TIF) [file ppat.1012991.s004.tif]

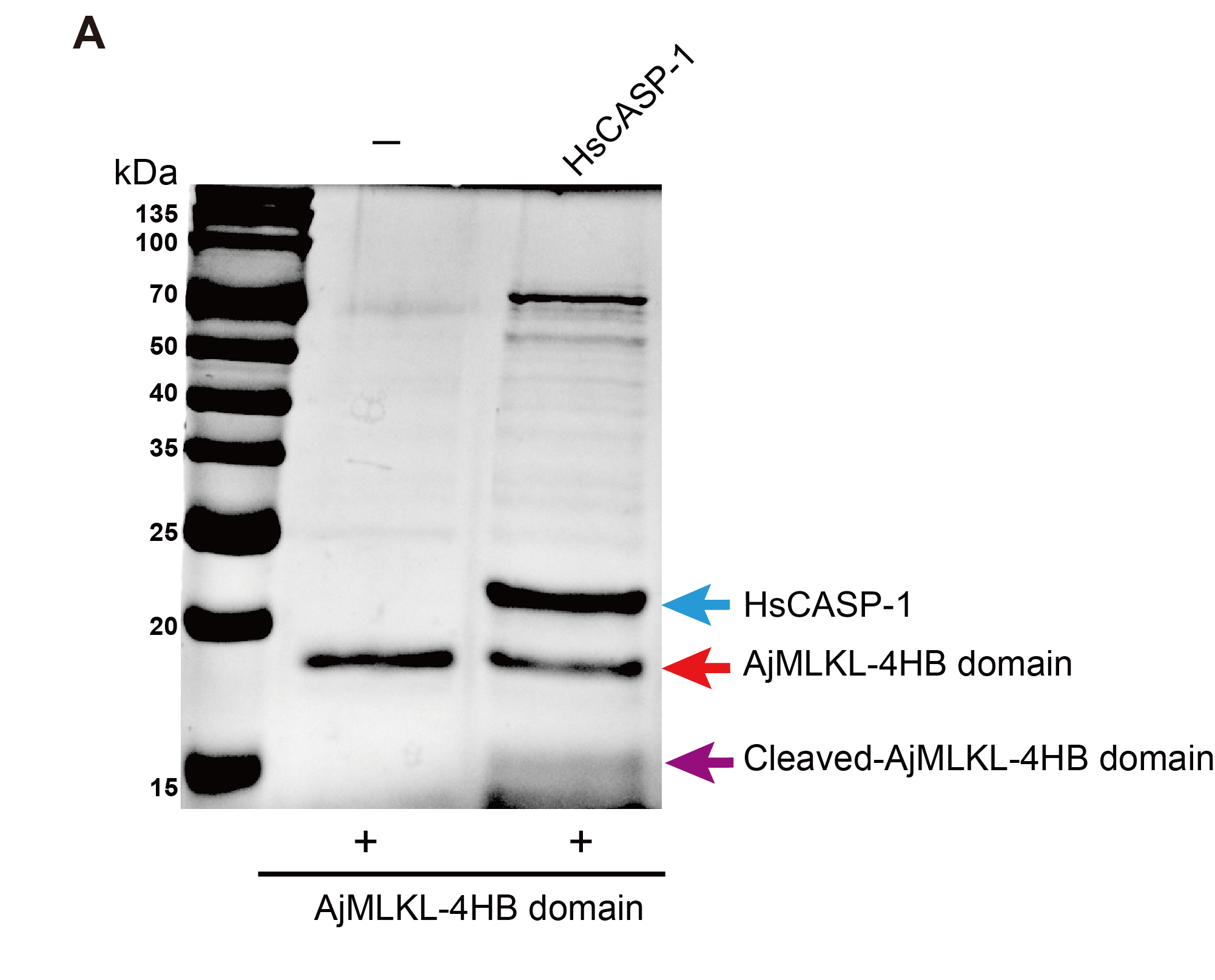

Supplement: S5 Fig — His-tagged AjMLKL-4HB was incubated with human caspases (HsCASP1) for 30 min and then stained with coomassie blue. (TIF) [file ppat.1012991.s005.tif]

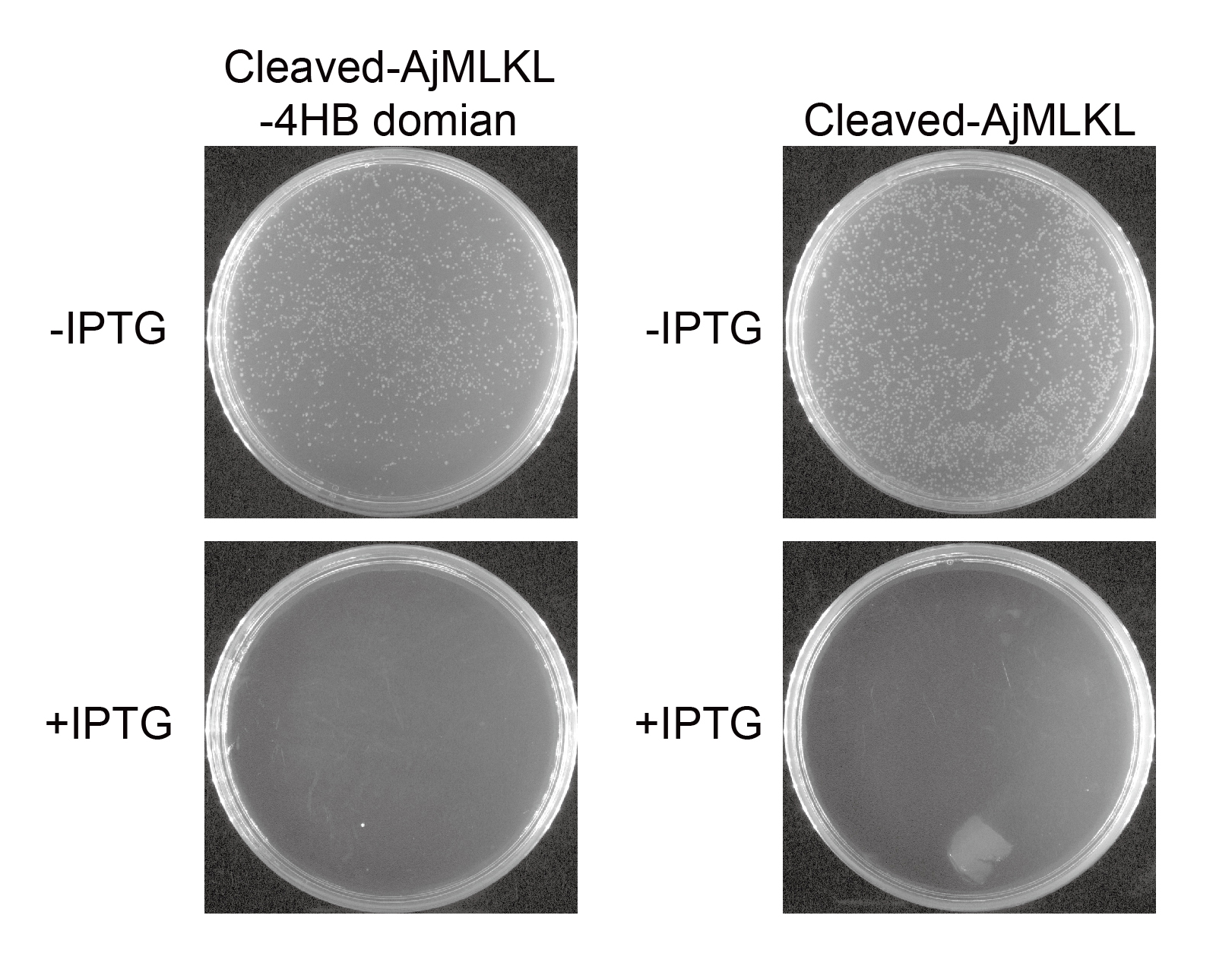

Supplement: S6 Fig — Escherichia coli expressing cleaved-AjMLKL-4HB (18-134 residues), cleaved-AjMLKL (18-491 residues) were grown on LB agar plates and induced with IPTG for 12 h at 16°C. (TIF) [file ppat.1012991.s006.tif]

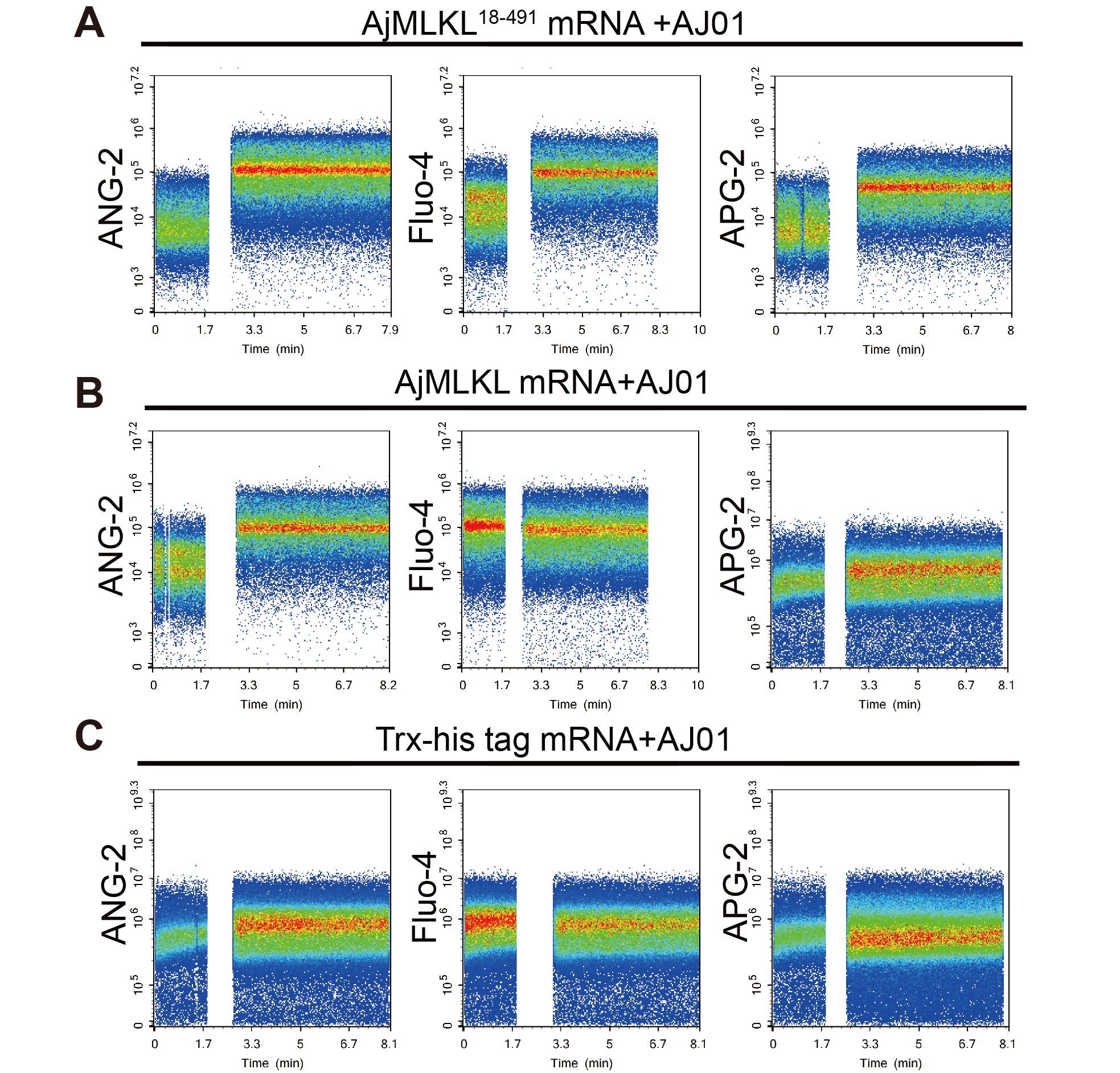

Supplement: S7 Fig — Intracellular ion concentration was monitored in uncleaved MLKL or cleaved MLKL overexpressed coelomocytes treated with AJ01. Sodium indicator (ANG-2), Calcium indicator (Fluo-4) and Potassium indicator (APG-2) were used. Trx-his Tag mRNA overexpressing coelomocytes were treated with AJ01 as a control. The coelocoelomocytes treated as above were collected and detected by flow cytometry. (TIF) [file ppat.1012991.s007.tif]

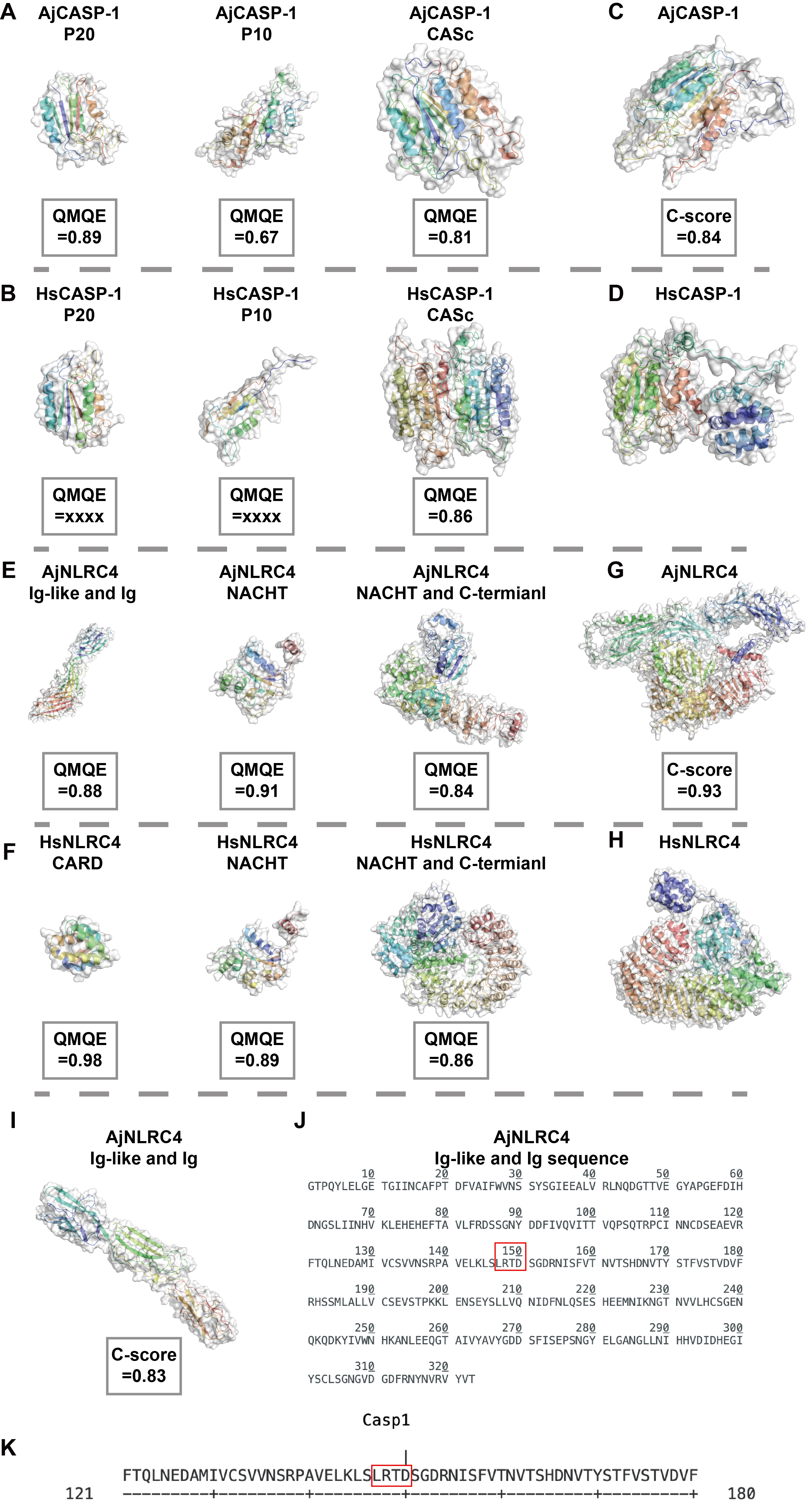

Supplement: S8 Fig — (A-D) Prediction of crystal structures of each domain of CASP-1 and tertiary structure of AjCASP-1 and HsCASP-1 by AlphaFold2 (E-H) Domain architecture and tertiary structure of AjNLRC4 and HsNLRC4 modeled by AlphaFold2. (I) Analysis of the structure of the Ig-like and Ig domain in AjNLRC4. The top five threading templates used by I-TASSER are 3alpA, 4fomA, 5zo1A, 6efzA, 1dgiR. (J and K) Amino acid sequences of the Ig-like and Ig domain of AjNLRC4, with the red box representing potential CASP-1 binding motifs. (TIF) [file ppat.1012991.s008.tif]

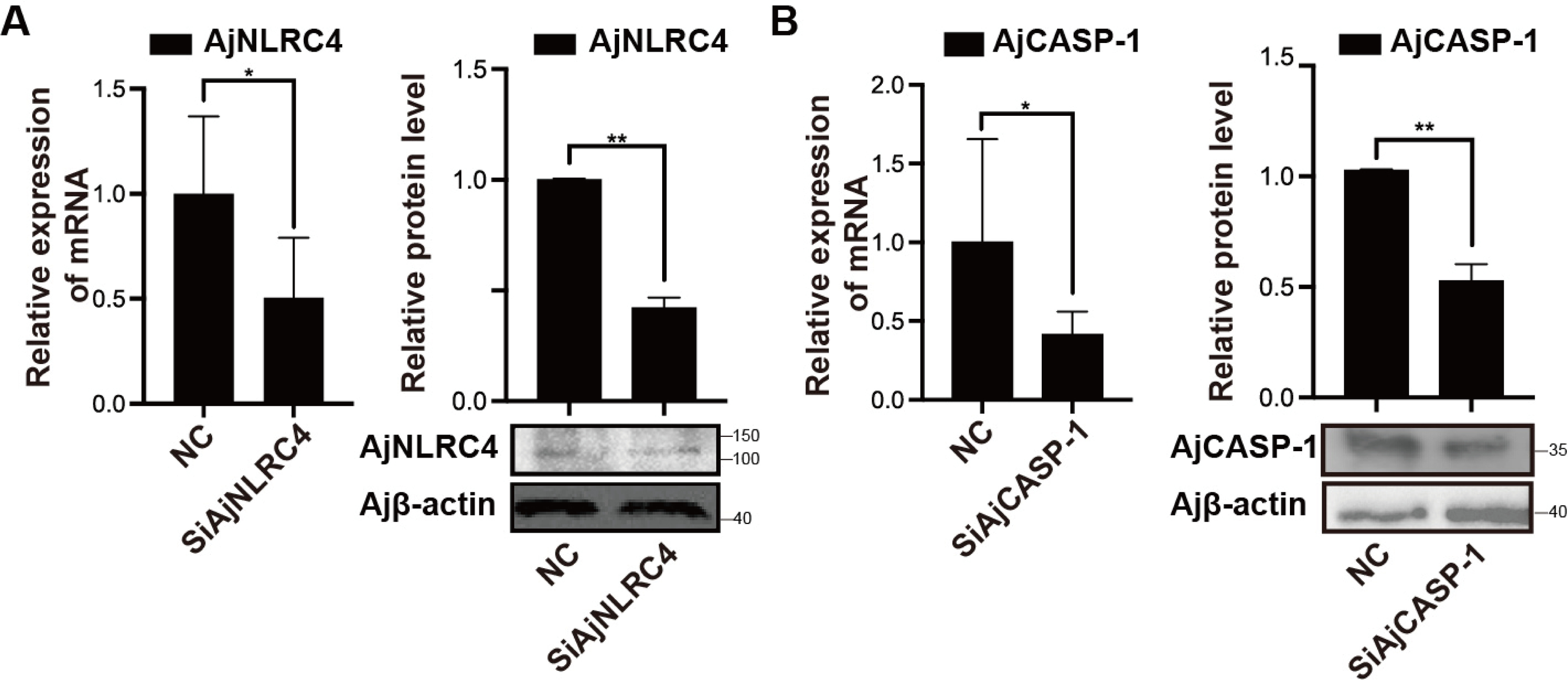

Supplement: S9 Fig — Sea cucumbers were transfected with specific AjNLRC4/AjCASP-1 siRNA for 24 h, respectively. The non-targeting siRNA (NC) was transfected as the control group. Then, 108 CFU/ml AJ01 at final concentration was added in the experimental and NC group for another 12 h. Finally, the relative expression of AjNLRC4 (a) or AjCASP-1 (b) in coelomocytes was determined at mRNA levels by using qPCR and at protein levels by western blotting analysis. It was found that after the above treatment, the mRNA and protein levels of AjNLRC4 were significantly decreased. Results are representative of at least three independent experiments, and error bars denote the SD of triplicate wells. *p < 0.05, **p < 0.01. (TIF) [file ppat.1012991.s009.tif]

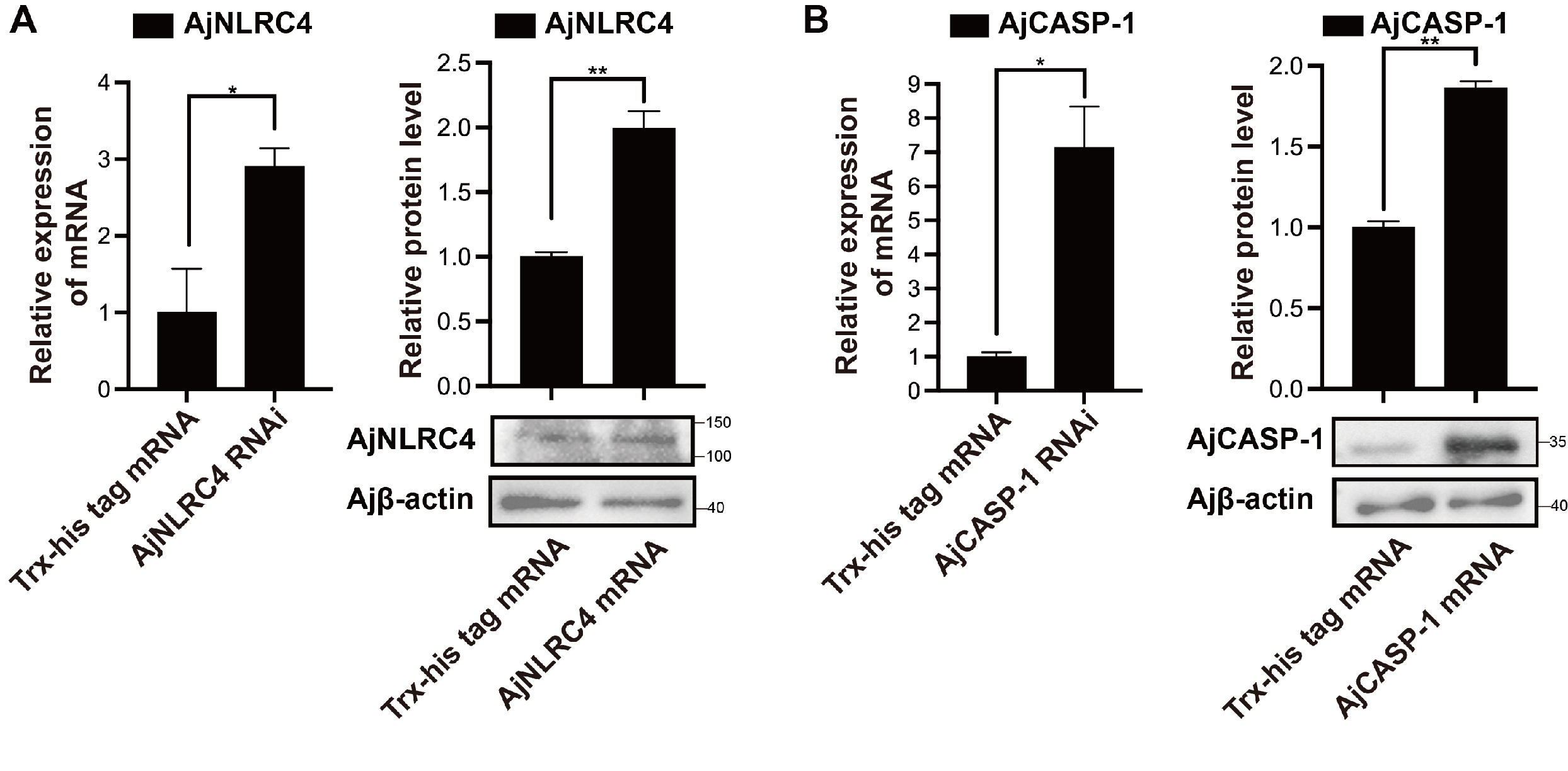

Supplement: S10 Fig — The Sea cucumbers were injected with AjNLRC4/AjCASP-1 mRNA for 24 h. The Trx-His tag mRNA was injected in as the control group. After the injection of AjNLRC4/AjCASP-1 mRNA, the mRNA and protein levels of AjNLRC4/AjCASP-1 mRNA were significantly up-regulated. Results are representative of at least three independent experiments, and error bars denote the SD of triplicate wells. *p < 0.05, **p < 0.01. (TIF) [file ppat.1012991.s010.tif]
