# Supplementary material for: Lytic coelomocyte death is tuned by cleavage but not phosphorylation of MLKL in echinoderms
Source: PLoS Pathog. 2025 Mar 14;21(3):e1012991. doi: 10.1371/journal.ppat.1012991 (PMC11932488; doi:10.1371/journal.ppat.1012991)
Supplement: S1 Table — (DOCX) [file ppat.1012991.s012.docx]

Table S1. Primers used in the study

| Primers | Sequences |
| --- | --- |
| Primers for siRNA |  |
| AjNLRC4-specific siRNA1 Sense | CCUUAUUACCUCAUCGAAUTT |
| RNA Interference Anti-sense | AUUCGAUGAGGUAAUAAGGTT |
| AjCASP-1-specific siRNA 1Sense | GAGGCUGACUUUCUUAUAATT |
| RNA Interference Anti-sense | UUAUAAGAAAGUCAGCCUCTT |
| AjCASP-1-specific siRNA 2Sense | GCGGCUCAAUGAUGAUGUATT |
| RNA Interference Anti-sense | UACAUCAUCAUUGAGCCGCTT |
| AjMLKL- specific siRNA Sense | GCUCUUAGGACUGAUAAAUTT |
| RNA Interference Anti-sense | AUUUAUCAGUCCUAAGAGCTT |
| siRNA-NC (Negative control) | UUCUCCGAACGUGUCACGUTT |
|  | ACGUGACACGUUCGGAGAATT |
| Primers for RT-PCR |  |
| AjNLRC4 qF | AAAGCCAATCTCGAAGAACAGG |
| AjNLRC4 qR | ACGAAAGTCGCCGTCAACAC |
| AjCaspase-1 qF | CGACTGCTTCTTGTGTGTGTTTC |
| AjCaspase-1 qR | AACTGATGTCTCAAGTGGCTCCT |
| AjMLKL qF | TAAAACTTGACGGATACGGGGAC |
| AjMLKL qR | TGTTCTGTCGGTTACCAATCTCA |
| Ajβ- actin qF | CCATTCAACCCTAAAGCCAACA |
| Ajβ-actin qR | ACACACCGTCTCCTGAGTCCAT |
| Primers for prokaryotic expression |  |
| AjNLRC4-EX-F *BamH* I | GGATCCGGGACGCCACAATACCTTGAGCTT |
| AjNLRC4-EX-R *Xho* I | CTCGAGGCAACCACTGACAACTGGAAACGC |
| AjNLRC4-IN *BamH* I | GGATCCGCAAGGATAATTCTTGAAGGGGAA |
| AjNLRC4-IN *Xho*1 | CTCGAGGACCACTGCCTTTCTGATATATGAA |
| AjCASP-1-ORF-F *BamH* I | GGATCCATGGCTGTAGCTGATATCCCATC |
| AjCASP-1-ORF-R *Xho* I | CTCGAGTCAAGTTTGGGCAGTAAGATGTA |
| AjCASP-1-C-F *BamH* I | GGATCCCGCTATGATATGCAACAACAGAC |
| AjCASP-1-C-R *Xho* I | CTCGAGTCAAGTTTGGGCAGTAAGATGTA |
| AjCASP-1-N-F *BamH* I | GGATCCATGGCTGTAGCTGATATCCCATC |
| AjCASP-1-N-R *Xho* I | CTCGAGTAAGTTGAAATGTATCGCTGACG |
| AjCASP-1-P20-F *BamH* I | GGATCC ACACGAGGTTTTGCTCACATTTTTA |
| AjCASP-1-P20-R *Xho* I | CTCGAG CGTTCCTCCACGACACGCTTGGATG |
| AjCASP-1-P10-F *BamH* I | GGATCC CCGACTGAGGCTGACTTTCTTATAA |
| AjCASP-1-P10-R *Xho* I | CTCGAG TCAAGTTTGGGCAGTAAGATGTAAC |
| AjMLKL-ORF-F *BamH* I | GGATCCATGGCAGGAGAAGTCACAGCAGC |
| AjMLKL-ORF-R *Xho* I | CTCGAGTTAGATTTCAACTCCTCCATTGCTC |
| AjMLKL-4HB domain-F *BamH* I | GGATCCATGGCAGGAGAAGTCACAGCAGTGA |
| AjMLKL-4HB domain-R *Xho* I | CTCGAGAACCACCTTCAAAATACCTTCCGGT |
| AjMLKL-STYKc domain-F *BamH* I | GGATCCCTTACTGATGAAACTGCCTTGGCAG |
| AjMLKL-STYKc domain-R *Xho* I | CTCGAGGATATCTGATACATTTGGTCTCTCT |
| Cleaved-AjMLKL-F *BamH* I | GGATCCGTCGGACTGAAAGCTTATAATGCAG |
| Cleaved-AjMLKL-R *Xho* I | CTCGAGTTAGATTTCAACTCCTCCATTGCTC |
| Cleaved AjMLKL-4HB domain-F *BamH* I | GGATCCGTCGGACTGAAAGCTTATAATGCAG |
| Cleaved-AjMLKL-4HB domain-R *Xho* I | CTCGAGAACCACCTTCAAAATACCTTCCGGT |
| Primers for MLKL mutations |  |
| MLKL^D17G^-F | CTATGTACGTTAGTAGCAGGTGTCGGA |
| MLKL^D17G^-R | CCTGCTACTAACGTACATAGATCAAT |
